# Understanding the Effects of Transcranial Electrical Stimulation in Numerical Cognition: A Systematic Review for Clinical Translation

**DOI:** 10.3390/jcm11082082

**Published:** 2022-04-07

**Authors:** Giulia Lazzaro, Elisa Fucà, Cristina Caciolo, Andrea Battisti, Floriana Costanzo, Cristiana Varuzza, Stefano Vicari, Deny Menghini

**Affiliations:** 1 Child and Adolescent Neuropsychiatry Unit, Department of Neuroscience, Bambino Gesù Children’s Hospital, IRCCS, 00146 Rome, Italy; giulia.lazzaro@opbg.net (G.L.); elisa.fuca@opbg.net (E.F.); cristina.caciolo@opbg.net (C.C.); andrea.battisti@opbg.net (A.B.); floriana.costanzo@opbg.net (F.C.); cristiana.varuzza@opbg.net (C.V.); stefano.vicari@opbg.net (S.V.); 2 Department of Human Science, LUMSA University, 00193 Rome, Italy; 3 Department of Life Science and Public Health, Università Cattolica del Sacro Cuore, 00168 Rome, Italy; 4 Centro di Riabilitazione Casa San Giuseppe, Opera Don Guanella, 00165 Rome, Italy

**Keywords:** number processing, arithmetic processing, cognitive training, non-invasive brain stimulation, interventions, dyscalculia

## Abstract

Atypical development of numerical cognition (dyscalculia) may increase the onset of neuropsychiatric symptoms, especially when untreated, and it may have long-term detrimental social consequences. However, evidence-based treatments are still lacking. Despite plenty of studies investigating the effects of transcranial electrical stimulation (tES) on numerical cognition, a systematized synthesis of results is still lacking. In the present systematic review (PROSPERO ID: CRD42021271139), we found that the majority of reports (20 out of 26) showed the effectiveness of tES in improving both number (80%) and arithmetic (76%) processing. In particular, anodal tDCS (regardless of lateralization) over parietal regions, bilateral tDCS (regardless of polarity/lateralization) over frontal regions, and tRNS (regardless of brain regions) strongly enhance number processing. While bilateral tDCS and tRNS over parietal and frontal regions and left anodal tDCS over frontal regions consistently improve arithmetic skills. In addition, tACS seems to be more effective than tDCS at ameliorating arithmetic learning. Despite the variability of methods and paucity of clinical studies, tES seems to be a promising brain-based treatment to enhance numerical cognition. Recommendations for clinical translation, future directions, and limitations are outlined.

## 1. Introduction

The successful development of numerical cognition—one of the most advanced cognitive abilities that humans possess—is crucial.

Numerical and arithmetic abilities are highly related to career options, overall living standards, and are equally important for life success as literacy [1,2]. Numerical cognition is becoming progressively relevant, with increasing focus on quantitative aptitude in occupational settings, and in the well-established pervasiveness of technology. 

On the other hand, atypical development of numerical cognition, such as dyscalculia, may increase the onset of neuropsychiatric symptoms, both internalizing and externalizing [3], especially when untreated. Interestingly, even when people do not present specific numerical cognition impairments, they may have everyday life problems in regard to manipulating numbers concurrent with specific anxiety symptoms—best known as *math anxiety* [4]. Math anxiety is an irrational emotional response, which includes tension, apprehension, or even dread, and it interferes with the ordinary manipulation of numbers and the solving of mathematical problems [4]. 

Numerical cognition impairment and its mental health-related consequences also imply significant public health expenditures—another variable not to be underestimated. For instance, in the United Kingdom, annual healthcare costs are estimated to equal nearly GBP 2.4 billion alone for numerical cognition difficulties [5]. 

With these premises, it is clear that an augmentation of numerical and arithmetic abilities could have a cascading effect on psychological levels as well as in the occupational and socioeconomic areas of people’s lives, supporting high qualities of life, well-being, and mental health. However, neurocognitive enhancement or interventional programs are still unrepresented, especially for individuals with dyscalculia. 

In recent decades, there has been a significant amount of research into the investigation of neurocognitive architecture associated with numerical cognition [6,7,8]. With the identification of specific cerebral networks, so-called *brain-directed* interventions have been employed to enhance certain aspects of numerical cognition. Transcranial electrical stimulation (tES) is one of the brain-directed techniques that has garnered academic and public attention. tES is an umbrella term that encompasses a range of tools used to manipulate (directly and non-invasively) brain activity and, in turn, modulate the related cognitive process or behavior [9]. tES is considered a painless and safe, user-friendly, cost-effective intervention [9]. 

Despite plenty of studies investigating the effects of tES on numerical cognition, a few non-systematic reviews have been published thus far, with some evidence in favor of such brain-directed techniques [10,11,12]. Four years ago, a meta-analysis by Simonsmeier et al. [13] demonstrated that tES improved learning more than performance. In the *stimulation of learning approach*, participants first participated in a learning intervention, e.g., they practiced mental arithmetic, and they received brain stimulation before or during the learning phase. After the learning phase, participants completed a learning outcome measure (e.g., to see how strongly their mental arithmetic competences improved) without brain stimulation [13]. In the *stimulation performance approach*, participants were assessed on a psychological construct (e.g., mathematical competence) before or during brain stimulation [13]. The meta-analysis comprised a small portion of published findings on numerical cognition (i.e., 12 studies)—including studies on language. However, more than a handful of studies have been published since 2018 and a comprehensive and systematized synthesis of results, specifically in the numerical cognition domain, is, thus far, still missing. In parallel, the need for interventional or neuroenhancement programs is increasing, especially for those who do not benefit from a first-choice treatment option, such as children and adults with dyscalculia [14,15]. 

To date, available interventions for dyscalculia mainly consist of educational strategies grounded in the use of concrete material and informational feedback to the learner and/or programs aimed at improving children’s numerical understanding—with some evidence of efficacy [16,17]. However, standardized and integrated evidence-based interventions for dyscalculia are still not available.

To the best of our knowledge, this is the first systematic review that comprehensively evaluates the potential positive effects of tES techniques on numerical cognition. In particular, a current systematic review would address the following research questions:

- Does tES consistently enhance numerical cognition?
- What are the numerical cognition aspects (i.e., number vs. arithmetic processing) in which tES techniques would be more effective?
- What tES technique would be more effective at ameliorating certain numerical cognition aspects? Under which stimulated brain regions?

Our systematic review aims to provide reliable knowledge for clinicians and researchers to prompt the acceleration of tES applications as promising neuroenhancements or treatment approaches in the numerical cognition field. 

To help the readers understand the results and conclusions, the introduction section will include a brief overview of the neurocognitive bases of numerical cognition—providing rationale for the brain networks targeted by tES studies—as well as a concise description of the basic principles of tES. 

### 1.1. Neurocognitive Bases of Numerical Cognition

**Figure 1:**
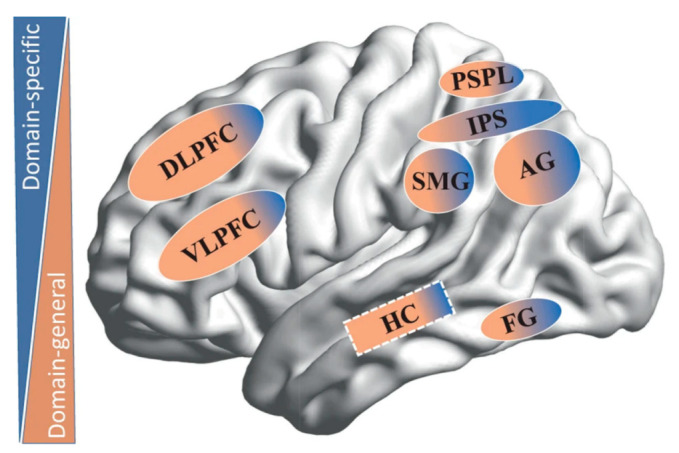
Neurobiological bases of numerical cognition. The neurocognitive bases are described as interactions of multiple brain networks—especially the fronto-parietal network—that supports domain-specific mechanisms (blue color coding) and domain-general processes (orange color coding). Legend: DLPFC = dorsolateral prefrontal cortex, VLPFC = ventrolateral prefrontal cortex, PSPL = posterior superior parietal lobe, IPS = intraparietal sulcus, SMG = supramarginal gyrus, AG = angular gyrus, FG = fusiform gyrus, HC = hippocampus. Reproduced from reference [18], 10.1038/s41539-021-00099-3, under the terms of the CC BY 4.0 license, http://creativecommons.org/licenses/by/4.0/ (accessed on 29 March 2022).

The involved neurocognitive bases are described as interactions of multiple brain networks that support domain-specific mechanisms on the one end, and domain-general processes on the other end (Figure 1) [11,18]. Regarding numerical cognition, domain-specific mechanisms are defined as mental operations that are exclusively related to certain aspects of basic number processing, such as the non-symbolic representation of numerical quantities. Domain-general processes are less specific to a particular domain, and mainly reflect mental operations that are important for learning and information processing, more generally, for example, working memory, visual–spatial reasoning, and attention (Figure 1) [11,18]. 

Here, we exclusively focus on the domain-specific mechanisms. This brief introduction describes *non-symbolic and symbolic number* *processes* and *arithmetic processes* mainly trained/assessed in numerical cognition paradigms combined with tES. Non-symbolic and symbolic number processes refer to a range of basic number abilities, such as automaticity in processing numerical information, the ability to discriminate and represent numerosity, and counting [19]. These skills are known to form the foundations for the acquisition of arithmetic processes, such as arithmetic number facts and the development of calculation skills [20,21,22]. The successful development of number processes predicts later school-based arithmetic achievements [23,24], and deficits in number and arithmetic processes have been described in individuals with dyscalculia [8]. 

#### 1.1.1. Non-Symbolic and Symbolic Number Processes 

Within non-symbolic processes, number sense has been widely studied and defined as the intuitive and innate ability to process numerical information without consciously dealing with symbolic representations of numbers [25]. This ability is shared among humans, encompassing newborns and indigenous tribes who have little or no formal mathematical education [26,27], as well as animal species; for reviews, see [28,29,30]. 

One subcomponent of number sense is the approximate number system (ANS, [23]), which allows for a quick, albeit inexact, non-symbolic estimation of the number of sensory objects and it supports intuitive judgments on numerical quantity. The ANS is usually measured or trained by numerosity discrimination paradigms that require the processing and comparing of non-symbolic quantities [23]. 

Even if under discussion [31], it seems that the refinement of the ANS into symbolic sophisticated number processes would represent a fundamental milestone in the development of numerical cognition. Within symbolic processes, the ability to represent a number on a horizontally oriented mental line—the so-called *mental number line*—is one of the most studied [32]. Strictly related to the concept of the mental number line is the ability to automatically process numbers, the so-called *numerical automaticity*—mainly assessed via symbolic number comparison tasks or the numerical Stroop task, which relies on the ability to process the magnitude of a number without effort [11]. 

On the neural level, non-symbolic and symbolic number processes rely on the posterior parietal cortex (PPC, [25,33,34,35,36]), specifically the bilateral intraparietal sulcus, and on the prefrontal cortex, basically the bilateral dorsolateral prefrontal cortex (dlPFC, [11]). Across development, a frontoparietal shift seems to occur. When processing numerical information, bilateral intraparietal sulcus and dlPFC are activated in younger children [35,37], while the involvement of left PPC increases along with the numerical processing refinement [38]. This functional and hemispheric specialization of the left intraparietal sulcus, along with the development of numerical processing, mirrors the automatization of this domain-specific process and, consequently, the reduced contribution of the domain-general processes along with the decreased activation of the dlPFC [11]. 

#### 1.1.2. Arithmetic Processes 

Non-symbolic and symbolic number processes contribute to the development of exact number skills. The *exact number system* is the system that allows precise representation, comparison, and manipulation of quantities with symbols [11]. The exact number system intervenes in numerical activities, such as (but not limited to) verbal counting or arithmetic processes, e.g., the retrieval of arithmetic facts from long-term memory and single or multidigit calculations (addition, subtraction, multiplication, and division [39]). 

The application of arithmetic processes involves the acquisition of two types of knowledge: *declarative knowledge* on arithmetic facts, allowing the direct retrieval of solutions to memorized arithmetic problems, and *procedural knowledge* about arithmetic operations, allowing the calculation of solutions to arithmetic problems. Arithmetic facts refer to simple arithmetic problems that have been stored in long-term memory and can be retrieved automatically without using computational strategies (e.g., immediately retrieving the fact that 5 + 4 = 9 without proceeding to any calculation [40]). Arithmetic facts rely on single-digit additions and multiplication tables [34] and they are crucial for the development of complex calculation abilities. Namely, the automatic retrieval of arithmetic facts allows the individual to allocate cognitive resources to the other processes required to solve complex calculations [8]. When arithmetic problems are not stored in long-term memory, individuals have to rely on written or mental calculations (e.g., computing that 35 + 21 = 56). Sophisticated computational skills leverage more on procedures (e.g., the ability to decompose the problem in multiple elements that are easier to manage) and, in turn, on domain-general processes, such as executive functions and working memory [8].

On the neural level, neuroimaging studies indicate large overlaps between brain regions for arithmetic processes and those for non-symbolic and symbolic numerical processes. However, besides the bilateral prefrontal cortex and the PPC, arithmetic tasks also recruit the temporo-parietal cortex (as the angular gyrus and the supramarginal gyri), and the medial temporal areas [6,41]. In particular, the activity in the temporo-parietal cortex has been associated with the retrieval of arithmetic facts from long-term memory and with mathematical expertise [42], reflecting the automatic mapping between an arithmetic problem and its answer. Further, the transition from a procedural counting strategy to memory-based retrieval systems is thought to be supported by a medial temporal lobe structure—the hippocampus—that seems to have a role in the initial consolidation of arithmetic facts in long-term-memory [43]. The prefrontal cortex is particularly involved in domain-general skills that are crucial during sophisticated computational tasks and when the solution cannot be retrieved from long-term memory [6]. Moreover, the bilateral intraparietal sulcus seems to be involved in numerical magnitude processing during subtractions, large problems, and in the execution of procedural strategies [6]. The left hemisphere of both parietal and frontal regions seems to be engaged during addition problems and the right hemisphere is dominant during multiplication problems [6].

### 1.2. Basic Principles of tES

tES is a portable and easy-to-use type of brain stimulation that allows to non-invasively modify brain activity and, in turn, behaviors [9]. 

Neuromodulation induced by tES is, per se, subthreshold to trigger neuronal firing, but it generates modifications in the neuronal threshold for depolarization, which can last after stimulation [44]. Since its mechanism of action is time-dependent, studies using tES are typically categorized into *online* or *offline* stimulation. Online tES refers to the application of the stimulation during a particular task or training, while offline tES is typically applied before the task or the training. During the online condition, tES can improve synaptic transmission if combined with a subthreshold stimulus [45], such as a task or training. When it occurs, the neural population is activated by the subthreshold stimulus and tES strengthens the cortical representation of the subserving cognitive functions [9]. The empowering effect derives from boosting synaptic strength in neural networks activated by concomitant activities. Therefore, the purpose of the combination of tES with a task/training is to promote the activation of neural networks underlying cognitive functions [46]. Concerning offline conditions, tES induces neuroplastic aftereffects via alteration of neurotransmitter activity [47]. 

The most known tES techniques comprise of—but are not limited to—the transcranial direct current stimulation (tDCS), high-definition tDCS (HD-tDCS), transcranial random noise stimulation (tRNS), and transcranial alternating current stimulation (tACS). To date, a new multifocal tES that combines tDCS and tACS, i.e., oscillatory direct current stimulation, is emerging [48,49]. However, only tDCS (and HD-tDCS), tRNS, and tACS are described in the present paragraph, in accordance with the technique applied in the reviewed studies.

**Figure 2:**
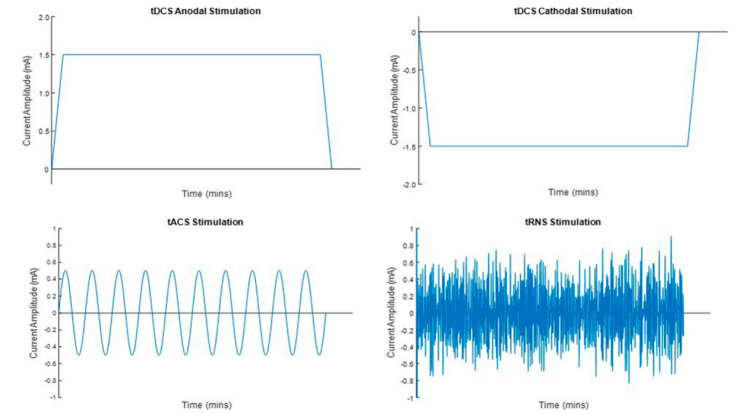
Stimulation waveforms for transcranial direct current stimulation (tDCS) (anodal and cathodal), transcranial alternating current stimulation (tACS), and transcranial random noise stimulation (tRNS). Reproduced from reference [59], https://doi.org/10.1007/s10545-018-0181-4, under the terms of the CC BY 4.0 license, http://creativecommons.org/licenses/by/4.0/ (accessed on 29 March 2022).

tDCS—the most widely used tES—involves the application of a low-amplitude direct current (0.5 to 2 mA, Figure 2) through at least one electrode (anode or cathode, usually 35 cm^2^) positioned above selected brain regions and a reference electrode. Its mechanism of action is polarity-dependent: anodal stimulation drives neural resting membrane potential closer to the activation threshold, increasing excitability; cathodal stimulation inhibits cell firing and decreases excitability [50]. In a newer form of tDCS—the HD-tDCS [51]—small HD electrodes are used instead of the two large sponge electrodes usually applied in the conventional tDCS. Compared to the previous approach, HD-tDCS has the advantage of giving much higher focality over the target region [52]. A typical montage for HD-tDCS is the 4 × 1-ring configuration, in which a central electrode is placed over the target region, and four return electrodes are placed around it in a ring-shaped configuration [53]. While anodal tDCS induces excitatory aftereffects that usually vanish 120 minutes post tDCS, the excitatory aftereffects of HD-tDCS steadily increase, with a peak of plastic-induced changes at 30 min post-stimulation and return to baseline at 6 h post-stimulation [52]. The resulting plasticity involves glutamatergic synapses and is calcium-dependent [54,55,56]. The long-lasting effects of tDCS are driven by several mechanisms that share some features with long-term potentiation and long-term depression [9]. 

Another tES that is sharply taking place is the tRNS. This polarity-independent form of tES involves the application of a weak current to the scalp throughout at least two electrodes at random intensities (i.e., ±0.5 mA) within a wide range of frequencies (from 0.1 to 640 Hz [50], Figure 2). tRNS acts via amplifying the effects of adding noise to a signal that is too weak to exceed a threshold on its own—a phenomenon called *stochastic resonance* [57,58]. However, the tRNS mechanism of action at the neural level is still under-debate [59]. Evidence shows that tRNS possibly boosts long-term potentiation-like cortical plasticity via inducing a repetitive opening of sodium channels shortening the hyperpolarization phase [57]. 

tACS is a still unexplored (and less used) tES. It generates an alternating current at a specific frequency (Figure 2), with the potential to synchronize or desynchronize activity between targeted brain regions through phase-locking and coherence mechanisms [60]. During a half cycle of stimulation, one electrode will function as an anode and the other one as a cathode, and current strength will increase and decrease following a half-sine wave. During the other half cycle, the pattern will reverse (the former anode should be considered as a cathode and vice versa), and the overall membrane potential is not affected [9]. Therefore, different from tDCS, which excites or inhibits cortical activity monotonously, the brain areas receiving stimulation during tACS are modulated in a similar way to each other. The effect of tACS depends on the cortical area being stimulated since the tACS potential to drive brain rhythms is higher when the externally superimposed oscillation is similar to the natural frequency of the cortical area being stimulated [61,62]. tACS aftereffects may rely on two main mechanisms: entrainment and spike-timing-dependent plasticity [63,64,65,66]. Entrainment refers to the synchronization of the endogenous brain oscillation to another exogenous driving frequency. It is thought to be most effective when the stimulated frequency is at or close to the endogenous frequency of the targeted brain regions. Whereas spike-timing-dependent plasticity refers to plastic changes that occur based on relative timing of the stimulated frequency to the endogenous frequency [66]. However, further research is needed to clarify the neurobiological mechanisms underpinning the effects of tACS since they are not yet well understood. 

## 2. Materials and Methods

### 2.1. Study Selection 

The study was conducted under the Preferred Reporting Items for Systematic Reviews and Meta-Analyses (PRISMA) recommendations, used for reporting systematic reviews and meta-analyses (see Table S1 in the Supplementary Materials [67]) and preregistered in the PROSPERO database, registration ID: CRD42021271139.

Study eligibility was assessed using the population, intervention, comparison, outcomes, and study design (PICOS) approach [68]. PICOS is a structured approach used to frame questions, using five components: the patient population or the disease being addressed (P), the interventions or exposure (I), the comparison group (C), the outcome or endpoint (O), and the study design chosen (S). To be included, studies had to fulfil the following criteria: (1) population: typically developing children and adolescents; healthy adults; healthy elderly individuals; individuals with numerical cognition difficulties (e.g., dyscalculia) of any ages; (2) interventions: tES (tDCS or HD-tDCS, tRNS, tACS) stand-alone or combined with numerical cognition training or tasks; (3) comparison group and outcomes: pre-/post- or during single/multiple sessions of real tES vs. sham tES (control condition) for one or more numerical cognition measures, including number processing and/or arithmetic tasks (accuracy, speed, efficiency); (4) study design: sham-controlled, randomized and non-randomized, blinded and non-blinded studies/clinical trials reporting accuracy, and/or speed and/or efficiency of numerical cognition measures as primary outcomes; (5) language: English written articles published in international peer-reviewed journals. No time restriction was applied to collect data from all studies published so far on the topic. Case reports and uncontrolled studies were excluded as well as reviews, proceedings abstracts, book contributions, and studies applying other types of stimulation (e.g., transcranial magnetic stimulation, vestibular stimulation). 

### 2.2. Search Procedure

Several strategies were used to identify the final study sample. First, databases “PubMed”, “Embase”, and “Scopus” were systematically searched without starting time restrictions, until 4 February, 2022, according to the following expressions: (“numerical cognition” OR “numerical learning” OR “number sense” OR “numerosity discrimination” OR “arithmetic learning” OR “arithmetic problem solving” OR “arithmetic procedures” OR “arithmetic fact retrieval” OR “mental calculation” OR “mathematical learning” OR “mathematical problem solving” OR “mathematical calculation”) AND (“transcranial direct current stimulation” OR “transcranial random noise stimulation” OR “transcranial alternating current stimulation” OR “transcranial electrical stimulation”). 

The first, second, and third authors conducted the literature search independently, screened the titles and abstracts of potentially eligible studies, examined the full texts, and extracted descriptive data, collaborating whenever the inclusion or exclusion of one study was doubtful. Second, the search was extended by identifying further studies from the references of the screened full texts. The final selection of articles was discussed by the first and last authors. The searches and the screening were run on Citavi 6 software. 

### 2.3. Data Extraction

The first, second, and third authors independently extracted data to confirm accuracy. Any doubt was discussed together with the help of the other authors. For each selected study, sociodemographic, sample, and methodological variables were extracted. Specifically, we extracted the following data:

(1) First author, country, and year of publication;
(2) Information of population characteristics: nonclinical (typically developing children, adolescents, and healthy adults and elderly individuals) and clinical (elderly individuals, adults, adolescents, children with numerical cognition difficulties) population; sample size; age (mean and standard deviation or age range, when provided); gender (males/females), handedness;
(3) Study design characteristics: randomized or non-randomized, blinded or non-blinded studies/clinical trials;
(4) tES protocol characteristics: type of tES (tDCS or HD-tDCS, tRNS, tACS); current intensity (mA) and frequency (Hz) when appropriate, i.e., tRNS or tACS; target electrode sizes (cm^2^); references electrode sizes (cm^2^); duration (in minutes); number of sessions; brain target and reference target; montage and conditions (bilateral; anodal, or cathodal) and conditions (real vs. sham);
(5) Timing of the task/training (offline, i.e., pre-/post- tES session(s) or online, i.e., during tES session(s));
(6) Outcomes and results: numerical cognition outcome(s), i.e., accuracy and/or speed (reaction times, RTs) and/or efficiency of training or tasks targeting non-symbolic and symbolic number and arithmetic processes.

## 3. Results

### 3.1. Study Selection

**Figure 3:**
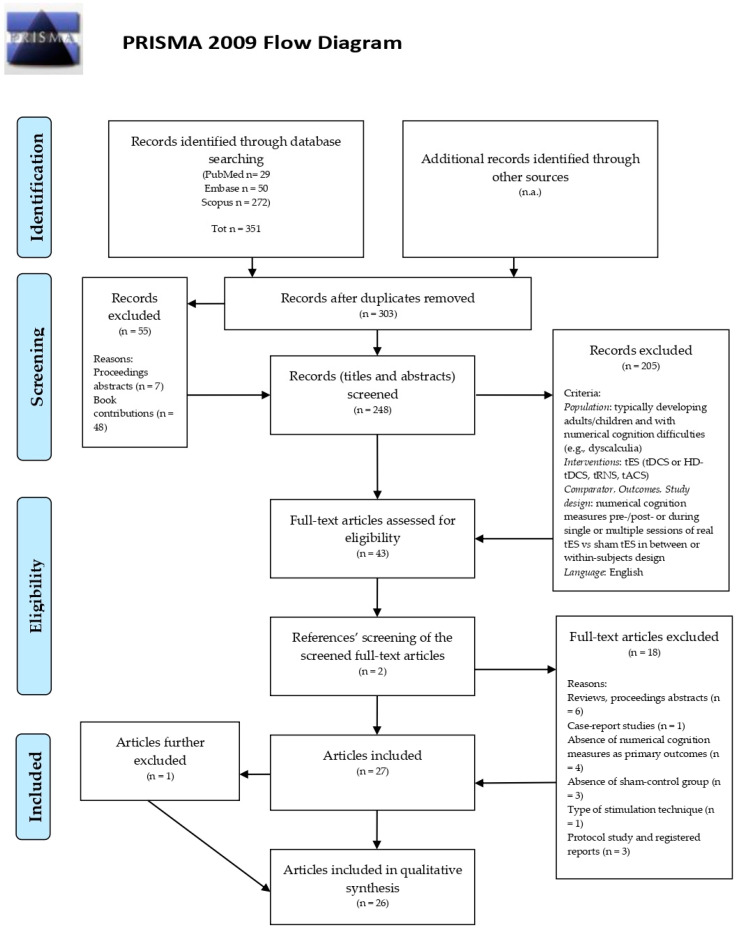
Search flow PRISMA diagram.

Figure 3 illustrates the detailed flowchart of the selection process. Database searching yielded 351 abstracts (PubMed: *n* = 29; Embase: *n* = 50; Scopus: *n* = 272). Of these, 48 were duplicates; after removing duplicates, a total of 303 abstracts remained. Of them, 55 abstracts were excluded since they did not meet the inclusion criteria (proceedings abstracts: *n* = 7; book contributions: *n* = 48). The remaining 248 titles and abstracts were examined for relevance and 205 were excluded. Two more records were found after screening the reference lists of the retrieved original articles. A total of 45 records were scrutinized, and 18 studies were excluded for the following reasons: reviews or proceedings abstracts (*n* = 6), case-report studies (*n* = 1), absence of numerical cognition measures as primary outcomes (*n* = 4), absence of sham-control group (*n* = 3), type of stimulation (vestibular stimulation, *n* = 1), and protocol study or pre-registered reports (*n* = 3). A total of 27 studies met the inclusion criteria. However, all studies except one, authored by Looi et al. [69], tested healthy participants. To maximize the interpretability of the results, we excluded the study. We consider the results and implications of the article by Looi et al. [69] in the integrative discussion section. A total of 26 studies were reviewed. Due to the heterogeneity in tES protocols and numerical cognition outcomes, a formal meta-analysis of results was not performed. 

### 3.2. Summary of Study Characteristics 

A summary of the reviewed tES studies according to the PICOS approach, together with the main results, are reported in Table 1 and Table 2. 

The 26 studies included in the present systematic review were conducted in 7 different countries: 12 in the United Kingdom, 5 in Switzerland, 4 in Germany, 2 in Austria, 1 in Turkey, 1 in Israel, and the last in Japan. The studies were published from 2010 to 2021. 

The sample size of included studies ranged from a minimum of 6 to a maximum of 137 participants (mean = 37.15; SD = 28.88). The studies constituted data from 936 healthy adults (age range: 18–60) and 30 healthy elderly individuals (age range: 60–73). None of the included studies involved adults with numerical cognition difficulties. There were 391 males and 537 females; only three studies did not indicate the male/female ratio. Of the total sample, 764 participants were right-handed and 46 were left-handed, while for the remaining 156 participants, the ‘handedness’ was not reported. 

Of the total works, 13 were designed as between-subjects studies (with a range of 1 to 6 sessions), and the remaining as within-subjects studies (2 crossover designs). 

The majority of studies applied tDCS and tRNS. Only one study used HD-tDCS while two studies applied tACS. Studies varied in tES parameters, such as current intensity (mA), electrodes size (cm^2^), polarization (i.e., tDCS), current frequency (i.e., tRNS, tACS), and current duration. 

Out of 26 studies, 9 studies investigated non-symbolic and symbolic number processes as primary outcomes, 16 studies investigated arithmetic processes, and 1 study considered both number and arithmetic processes as primary outcomes. To provide a clear description of the selected sample of studies, records were divided based on the targeted numerical cognition processes (i.e., non-symbolic and symbolic number processes, and arithmetic processes). Results were described according to the type of tES within non-symbolic and symbolic number processes and arithmetic processes.

**Figure 4:**
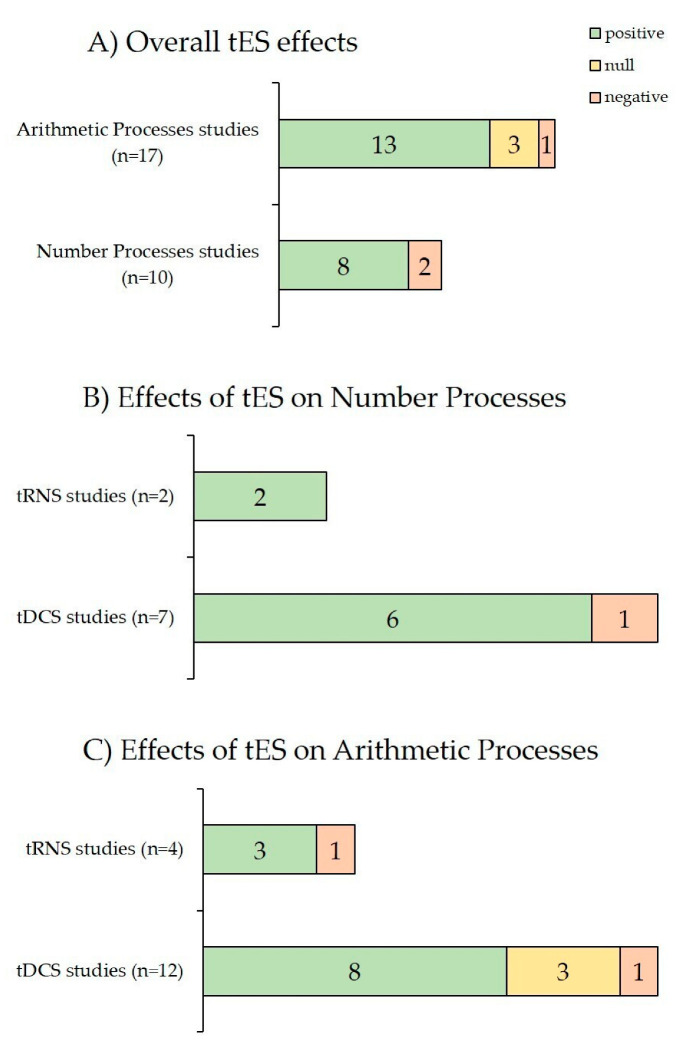
Summary of tES effects. Panel (**A**) shows the tES effects across all reviewed studies based on non-symbolic and symbolic number processes and arithmetic processes. Panel (**B**) shows the comparisons between tDCS and tRNS effects across all studies addressing non-symbolic and symbolic number processes. Panel (**C**) shows the comparisons between tDCS and tRNS effects across all studies addressing arithmetic processes. The effects of HD-tDCS were included in the tDCS effects. The effects of tACS were also not considered since only one study was found. Legend: tES = transcranial electrical stimulation; tDCS = transcranial direct current stimulation, tRNS = transcranial random noise stimulation.

Refer to Figure 4 (panel A) for a visualization of the qualitative effects of tES techniques across all reviewed studies based on non-symbolic and symbolic number processing (positive results: 8 out of 10 reports) and arithmetic processing (positive results: 13 out of 17 reports).

### 3.3. Non-Symbolic and Symbolic Number Processes

Concerning the studies reported on in this paragraph (see Table 1), 6 out of 10 reports evaluated the tES effects on tasks (stimulation performance approach [70,71,72,73,74,75]. Cohen Kadosh et al. [70] also administrated training, but only pre- and post-task performances were reported). Three studies involved tasks and training (stimulation learning and performance approaches [76,77,78]), and the remaining one was only on training (stimulation learning approach [79]). To facilitate the reading of results, a detailed description of the paradigm (tasks and trainings) used in the selected tES studies is given in Box S1 (see Supplementary Materials). 

Concerning the timing of administration of tES in relation to the training/tasks, half of the studies (5 out of 10 [74,76,77,78,79]) assessed numerical cognition outcomes both online and offline, 3 of the studies only online [72,73,75], and the remaining 2 only offline [70,71]. 

Among the five studies administrating training, four studies included a follow-up assessment (2 months later [79]; 4 months later [77,78]; 6 months later [70]). Moreover, four studies evaluated tES effects on untrained skills or to other numerical or arithmetic processes, which shared part of the stimulated brain network—known as *transfer effects* [70,76,77,78]. 

#### 3.3.1. tDCS

In our systematic review, the first study that applied tES to enhance numerical cognition in healthy adults was conducted by Cohen Kadosh et al. [70]. This between-subjects, randomized, single-blind study combined 6 days of tDCS to bilateral PPC with *artificial symbols training* (see Box S1, part A) to simulate the acquisition of automatic number processing—known as the *numerical learning process*. The training involved learning the implicit associations between meaningless artificial symbols and their corresponding, arbitrarily-assigned, numerical magnitudes. After each session, participants were assessed on their capacities to automatically process these symbols—i.e., numerical automaticity—using the numerical Stroop paradigm and the number line representation of the acquired artificial digits (see *artificial digits version* of *numerical Stroop task* and *number line task*, Box S1, part B). Results showed that left-cathodal/right-anodal (LC-RA) tDCS combined with the numerical learning paradigm determined better (and more consistent) numerical automaticity at the end of the training (increased numerical Stroop and number line performance) compared to the sham and left-anodal/right-cathodal (LA-RC) tDCS. Moreover, the effect was long-lasting and persisted at the 6-month follow-up. Interestingly, the effect was observed in the trained symbols (i.e., artificial digits version of the numerical Stroop and number line tasks) but not in the control tasks (i.e., *Arabic digits* version of the *numerical Stroop* and *number line tasks*, Box S1, part B). It means that no transfer effects were found, because the effect of stimulation was specific to the representation of the trained symbols. 

Another study involving healthy adults [76] included the same experimental design with the addition of the bilateral dlPFC as a stimulation target. The effect of 6-days of tDCS on numerical learning (trained by the artificial symbol training), and on numerical automaticity (assessed by the artificial digits version of the numerical Stroop task) was investigated. Results showed that LA-RC tDCS over bilateral dlPFC decreased the speed of the overall numerical learning rate acquisition, but enhanced the numerical automaticity, as indicated by a larger numerical Stroop effect (compared to sham and LA-RC tDCS over bilateral PPC). Instead, LA-RC tDCS over bilateral PPC produced the opposite effects, promoting the highest numerical learning rate acquisition and reducing the numerical automaticity (compared to sham and LA-RC tDCS over bilateral dlPFC). As in the previous study [70], the numerical automaticity increment was referred only to the trained materials, supporting again the specificity of tDCS effects. 

In the same year, Hauser et al. [71], in a series of within-subjects, single-blind experiments with a group of healthy adults ( for convenience, the study is first described in the non-symbolic and symbolic number processes paragraph, and will not be reported on in the arithmetic processes paragraph, to avoid weighing down the reading of the results) tested the effects of a single-session of left-anodal (LA) tDCS, right-anodal (RA) tDCS, bilateral anodal (BA) tDCS, bilateral cathodal (BC) tDCS, and sham tDCS over PPC in the symbolic number and arithmetic processes. Results showed that LA tDCS over PPC was the most effective configuration to improve both number and arithmetic processes compared to sham tDCS, as measured by the enhanced accuracy in the *double-digit number comparison task* (see Box S1, part B) and the fostered RTs in the *double-digit subtraction* task (see Box S2, part B). It suggests that excitatory stimulation over left parietal regions would have an impact on both numerical and arithmetic processing. 

In line with the aforementioned findings, two years later, Li et al. [72], in a within-subjects, single-blind study, investigated the effects of a single-session of LC-RA tDCS over PPC, LA-RC tDCS over PPC and sham tDCS, while a group of healthy adults performed a *number comparison task* (see Box S1, part B)—a task similar to that administrated by Hauser et al. [71]. Results demonstrated that LC-RA tDCS over PPC worsened participants’ performances in the number comparison task compared to sham tDCS by slowing RTs. It seems to reinforce the notion that only excitatory stimulation over left parietal regions would be the best option to improve number processing since the authors found a worsened effect during inhibitory/excitatory stimulation over left/right parietal regions. 

Brezis et al. [73], in the following year, found a contrasting result. In a within-subjects, single-blind study, the authors explored the effects of a single-session of RA tDCS over PPC, RA tDCS over dlPFC, and sham tDCS during a *numerical averaging task* (see Box S1, part B) in a group of healthy adults. The results indicated that the effect of RA tDCS over PPC was higher than the effect of sham and that sham and RA tDCS over dlPFC did not differ. 

Looi et al. [79], in the same year, investigated whether 2 days of LC-RA tDCS over bilateral dlPFC combined with an adaptive number line training on mapping fractions (see Box S1, part A) would enhance the numerical performance compared to sham tDCS, as well as promote long-lasting effects. The results of this between-subjects, single-blind, randomized study showed that LC-RA tDCS over bilateral dlPFC significantly supported the fractions mapping training compared to sham tDCS (as indicated by the decrease of RTs and improved accuracy, even at the most difficult precision). In line with a previous study on tDCS [70], the effects persisted even after a 2-month follow-up. 

Given the differences between conventional tDCS and HD-tDCS in terms of set-up, focality, and aftereffects, the only study using HD-tDCS will be described in the following paragraph. 

#### 3.3.2. HD-tDCS

Hartmann and collaborators [74], in a within-subjects, counterbalanced, single-blind study, submitted a group of healthy adults to a single-session of LA HD-tDCS, RA HD-tDCS, and sham HD-tDCS over PPC while performing a *non-symbolic approximate arithmetic task* (i.e., additions and subtractions, see Box S1, part B). Results documented that, in terms of accuracy, HD-tDCS over PPC (regardless of laterality) improved online non-symbolic approximate subtraction accuracy compared to sham HD-tDCS. Specifically, while there is a general tendency to underestimate the results of subtraction problems—known as the operational momentum effect—the tendency to underestimate the subtraction results after HD-tDCS over PPC was reduced. These results are in line with those of Hauser et al. [71] and Brezis et al. [73], demonstrating that only unilateral left and right excitatory stimulation over PPC would enhance number processing. In fact, bilateral parietal tDCS (as in [72,76]) seems to produce contrasting and confounding evidence, probably depending on the processes intended to modulate or the combined tasks. 

However, a significant effect for speed–accuracy trade-off was not found. Similarly, no significant difference was found for online additions and offline additions and subtractions, in terms of accuracy or RTs. 

#### 3.3.3. tRNS

Cappelletti et al. [77], in a between-subjects, randomized, double-blind study, investigated the long-term effects of 5 days of tRNS coupled with *numerosity discrimination training* (see Box S1, part A; the study is described in the non-symbolic and symbolic number processes paragraph because its primary outcome was the effect on a non-symbolic number process and arithmetic processes were investigated only to assess the potential transfer effects as secondary outcomes). The numerosity discrimination paradigm assesses the ability to discriminate numerosity in terms of number acuity, which is thought to rely on the ANS [23]. Four groups of healthy adults were compared on number acuity: real and sham tRNS over bilateral PPC coupled with numerosity discrimination training, tRNS over motor areas coupled with numerosity discrimination training (control sites-group), and a passive control group that received tRNS over bilateral PPC without numerosity discrimination training. Although at post-test, each group improved in number acuity, a significantly better performance from baseline was found when the training was coupled with tRNS over bilateral PPC compared to the other conditions (sham tRNS, tRNS over motor areas, and tRNS without training). Nevertheless, the four groups did not differ on tasks linked to number acuity, as the *numerical Stroop task*, the *non-symbolic approximate arithmetic task* (see Box S1, part B), and *arithmetical processing task* (see Box S2, part B). It again means that enhancing training through stimulation would have little chance of near transfer effects. However, the improvement observed in the training after the tRNS over bilateral PPC persisted also at the 4-month follow-up, while the performance declined over time in the sham tRNS. In turn, the effect on the number acuity was maintained at long-term only when training was combined with stimulation over parietal regions.

Cappelletti et al. [78], using the same numerosity discrimination paradigm but without a passive control group, confirmed their previous results in a sample of healthy adults and elderly individuals. The authors found that 5 days of tRNS over bilateral PPC significantly enhanced (from baseline) the effects of the numerosity discrimination training compared to sham and motor tRNS in both adults and the elderly. As previously found [77], the positive effect on numerosity discrimination persisted at the 4-month follow-up (but did not affect other tasks, such as the non-symbolic approximate arithmetic task, numerical Stroop task, and arithmetical processing task). Similar to previous results, the stimulation over parietal regions increased in number acuity; the effect was maintained in the long-term but no near transfer effects have been shown. 

#### 3.3.4. tACS

Labree et al. [75] recently investigated the causal link between inhibitory control and numerosity discrimination abilities via parietal alpha oscillations in two experiments. In the first within-subjects, counterbalance, double-blind experiment, the authors submitted a group of healthy adults to a single-session of theta-tACS over PPC, alpha-tACS over PPC, beta-tACS over PPC, and sham-tACS over PPC while performing a *numerosity discrimination task* (see Box S1, part B). Results showed that alpha-tACS over PPC significantly and specifically worsened performance on the numerosity discrimination task (only in incongruent trials) compared to the other conditions. The authors explained that such detrimental effects could be possible due to desynchronization of parietal neuronal oscillations in the alpha range. However, to further confirm the causal and specific involvement of parietal brain oscillations, a group of healthy adults participated in a second within-subjects, counterbalanced, single-blind experiment with control-site conditions. Participants underwent single-sessions of alpha-tACS over PPC, of alpha-tACS over dlPFC and the corresponding sham conditions while performing a numerosity discrimination task. The results further confirm the worsening effect of alpha-tACS over PPC compared to sham alpha-tACS, while no difference was found between alpha-tACS over dlPFC and the corresponding sham condition. Overall, the experiments confirmed the hypotheses that parietal alpha oscillations—an electrophysiological index of inhibitory abilities—are linked to numerosity and reinforced the view that these abilities are intrinsic to numerosity discrimination.

Figure 4 (panel B) summarizes and compares the effects of tDCS and tRNS in number processing. Six out of seven tDCS studies reported beneficial effects, as well as the two tRNS studies. Null effects were not reported overall, so they were not represented. Effects of HD-tDCS were included in tDCS effects. The effects of tACS were also not considered since only one study was found. 

### 3.4. Arithmetic Processes 

Concerning studies reported in this paragraph (Table 2), 12 out of 16 reports evaluated tES effects on tasks (stimulation performance approach [80,81,82,83,84,85,86,87,88,89,90,91]), and the remaining studies only on training (stimulation learning approach [92,93,94,95]). To facilitate the reading of the results on arithmetic processes, a detailed description of the paradigm (tasks and trainings) used in the selected tES studies is reported in Box S2 (see Supplementary Materials). Concerning the timing of administration of tES in relation to the training/tasks, half of the studies (8 out of 16: [81,87,88,91,92,93,94,95]) assessed numerical cognition outcomes both online and offline, 6 studies only online [82,83,84,86,89,90], and the remaining 2 studies only offline [80,85]. Among the studies, three reports included a follow-up assessment (24 h later [93]; 7 days later [88]; 6 months later [92]). Moreover, four studies evaluated transfer effects [88,92,93,94].

#### 3.4.1. tDCS

Clemens et al. [80], in a within-subjects, counterbalanced, single-blind study, examined whether a single-session of RA tDCS over the angular gyrus of PPC would modulate the performances in a group of healthy adults. Results showed that one session of RA tDCS over PPC did not improve arithmetic fact retrieval (*simple multiplication verification task*, see Box S2, part B) compared to sham tDCS and a control active group without electrode implementation.

Klein et al. [82], that same year, in a within-subjects, counterbalanced study, investigated whether a single-session of left-anodal/right-anodal (LA-RA) tDCS, left-cathodal/right-cathodal (LC-RC) tDCS, and sham tDCS over PPC would improve arithmetic calculations in a group of healthy adults while they performed an *addition task* (see Box S2, part B). Findings documented that one session of LA-RA tDCS over PPC improved addition calculation performances (in terms of RTs) compared to sham tDCS and LC-RC tDCS. 

Together, these results confirm the variability of tDCS outcomes, which strongly depend on hemispheric lateralization of the engaged processes. 

Kasahara et al. [81], in line with this consideration, in that same year, in a crossover within-subjects, single-blind design with healthy adults, explored whether individual differences in the functional lateralization of brain activity might modulate tDCS effects. First, parietal activity lateralization was evaluated by using fMRI during a *mental calculation task* (see Box S2, part B). Secondly, participants performed the mental calculation tasks before, after tDCS (30 min and 60 min post-tDCS), and online, while they received LA-RC tDCS, LC-RA tDCS, and sham tDCS over bilateral PPC. Interestingly, LA-RC tDCS over bilateral PPC improved calculation performance (i.e., faster RTs) only in participants with parietal left-hemisphere dominance, compared to LC-RA tDCS and sham tDCS. However, tDCS did not significantly affect the performance of participants with bilateral parietal activation. A direct comparison between the left-hemisphere dominance group and the bilateral activation group on calculation performance revealed that RTs in the LA-RC tDCS session was shorter in the left-hemispheric dominance group than in the bilateral activation group. This study suggests that hemispheric lateralization, or more in general, individual differences, markedly influence tDCS effects. 

Sarkar et al. [83] conducted a crossover, within-subjects, double-blind study that demonstrated the influence of individual differences in tES outcomes in healthy adults. Results showed that LC-RA tDCS over bilateral dlPFC modulated performances on a *simple arithmetic decision task* (see Box S2, part B) based on the mathematical anxiety of participants. Participants with higher mathematical anxiety were faster at arithmetic decisions (i.e., lower RTs) after LC-RA tDCS over bilateral dlPFC, while participants with lower mathematical anxiety were slower in arithmetic decisions (i.e., higher RTs) compared with the sham stimulation. In sum, individual traits, such as mathematical anxiety, could affect brain stimulation outcomes. 

One year later, Grabner et al. [93], in a between-subjects, randomized, double-blind study with healthy adults, evaluated the effects of LA tDCS, LC tDCS, and sham tDCS over PPC during short-term arithmetic training (i.e., arithmetic learning) by using *complex multiplication and subtraction problems* (see Box S2, part A). The stability of the stimulation-induced learning effects was assessed in a follow-up test (i.e., arithmetic automaticity) the day after the training. Results revealed that even within such a short period of stimulation, tDCS over PPC affected the arithmetic learning progress. During the learning session, LA tDCS selectively promoted significant improvement in accuracy for subtractions but not multiplication learning compared to sham and LC tDCS. However, these effects were not maintained 24 h later and were not transferred in automaticity. Moreover, LC tDCS reduced the learning rate (in terms of RTs) of both complex multiplication problems and subtraction problems compared to sham and LA tDCS. These negative effects were maintained 24 h post-stimulation only for trained problems, indicating that LC tDCS would significantly worsen automaticity effects.

Rütsche et al. [86], in a within-subjects, single-blind study in healthy adults, explored the effects of a single session of LA tDCS on the complexity of arithmetic tasks (*small vs. large additions and subtractions*, Box S2, part B). LA tDCS over PPC enhanced the performance in large problems in terms of RTs but decreased the performance in small problems in terms of accuracy compared to sham tDCS. These results suggest that tDCS effects are more effective under demanding and engaging conditions. 

Pope et al. [84], in line with this view, in a between-subjects, randomized, single-blind study, investigated whether LA tDCS over dlPFC could improve performance when cognitive demands were high. A group of healthy adults performed two arithmetic tasks, the *Paced Auditory Serial Addition Task* (*PASAT*, see Box S2, part B) and the demanding *Paced Auditory Serial Subtraction Task* (*PASST*, see Box S2, part B). Results confirmed the expectations, because the performance (in terms of accuracy and RTs) was significantly better in the PASST after LA tDCS compared to LC tDCS and sham tDCS. 

In the same year, Artemenko et al. [85], in a within-subjects, counterbalanced study in healthy adults, evaluated the effects of right-cathodal (RC) tDCS over PPC, RA tDCS over PPC, LC tDCS over PPC, LA tDCS over PPC and sham tDCS while participants performed an *addition task* (see Box S2, part B). Overall, results showed no main effects of stimulation conditions in the addition calculation, in terms of RTs. 

In the following year, Hauser et al. [87] found a converging null tDCS effect. In a between-subjects, randomized, double-blind study in healthy adults, the authors investigated the effects of LA-RC tDCS over bilateral PPC and sham tDCS during two complex subtraction problems (*arithmetic facts retrieval and calculations*, Box S2, part B). Results showed no effects of stimulation regardless of condition (real vs. sham) or task (arithmetic facts retrieval vs. calculations). 

Recently, Mosbacher et al. [91], in a between-subjects, double-blind study in healthy adults, investigated the effects of LA tDCS over dlPFC, LA tDCS over PPC, and sham tDCS during and after an arithmetic task (*small* vs. *large additions and subtractions*, Box S1, part B). Results revealed that, in the large subtractions problems, LA tDCS over dlPFC significantly improved performance from baseline (i.e., faster RTs) during and after stimulation. However, large subtractions did not improve during sham tDCS but only after the stimulation. No effect was observed in the LA tDCS over PPC nor in the other arithmetic problems (small additions, large additions, and small subtractions). 

In the following year, Mosbacher et al. [95] explored the effects of six active conditions (LA tDCS over dlPFC, LA tDCS over PPC, alpha-tACS over dlPFC, alpha-tACS over PPC, theta-tACS over dlPFC, theta-tACS over PPC) and a sham tDCS or tACS group on the acquisition of arithmetic procedures (*arithmetic learning task*, see Box S2, part A). In this between-subjects, randomized double-blind study, results showed that theta-tACS over dlPFC reduced the repetitions needed to learn novel facts compared to the sham group. Moreover, both theta-tACS over dlPFC and PPC accelerated the calculation speed in fact learning problems. 

#### 3.4.2. tRNS

Snowball et al. [92], in a between-subjects, randomized, double-blind study, first assessed the combined effects of 5 days of tRNS over bilateral dlPFC combined with cognitive training in healthy adults. Specifically, the cognitive training was based on a *drill learning training* (Box S2, part A) and *calculations learning training* (Box S2, part A). Results showed that tRNS over bilateral dlPFC significantly enhanced the learning rate on both drill and calculation tasks compared to sham tRNS. Only the effect on calculation was evident at the 6-month follow-up (i.e., faster RTs). Further, there was some evidence of transfer, with enhanced performance on untrained calculation problems at the follow-up only in participants who underwent tRNS. 

Popescu et al. [94], using these previous results, in a between-subjects, randomized, double-blind study with healthy adults, examined whether 5 days of tRNS would facilitate drills and calculation learning to sham tRNS, using a different electrodes montage. Different from Snowball et al. [92], participants received stimulation over bilateral dlPFC for the first 3 days and over bilateral PPC for the remaining 2 days of training. Task difficulties varied and were determined by the size of the problem set: in the “easy” condition, there were fewer but more frequently repeated problems than in the “difficult” condition, in which there were more, and fewer repeated problems. While no significant effect was found for drills, RTs were significantly faster for calculations during tRNS compared to sham tRNS only in the difficult condition. No effect was found for accuracy. Moreover, after the end of the 5 days of training, no effects were found for drills. Regarding calculations, the effects after the end of the 5 days of training on RTs were similar to the one obtained during tRNS with faster performances for difficult conditions compared to sham tRNS for both trained and untrained problems. After the end of the training, an effect was also found for accuracy with reduced accuracy for the untrained problems during sham tRNS compared to tRNS. It was also demonstrated that, for the participants in the sham group, calculations with untrained problems were worse when the training was less effortful.

In the same year, Pasqualotto [88], in a between-subjects, randomized, double-blind study with healthy adults, investigated whether a single session of tRNS (over bilateral dlPFC or bilateral PPC) during a *subtraction verification task* (see Box S2, part B) would improve performance compared to the sham. Results suggested a significant reduction of RTs during the tRNS over bilateral dlPFC and bilateral PPC compared to sham tRNS. However, after 7 days, no effects were found in the RTs and between trained and untrained problems. Regarding accuracy, no effects were evident during the stimulation conditions, but a significant increment emerged after 7 days between tRNS (over bilateral dlPFC or bilateral PPC) compared to the sham. 

Two years later, Bieck et al. [89], in a within-subjects, counterbalance, single-blind study, studied whether a single-session of tRNS over PPC, tRNS over dlPFC, sham tRNS over PPC, and sham tRNS over dlPFC modulated arithmetic processing while participants performed an addition task (see Box S2, part B). Results showed a significant but light improvement during tRNS over dlPFC compared to tRNS over PPC and sham conditions. 

In the last few years, Krause et al. [90], in a within-subjects, randomized, double-blind study, investigated the effects of a single session of tRNS over bilateral dlPFC in six mathematically highly-proficient, healthy, postgraduate students during a *complex calculation task* (see Box S2, part B). During the sham condition, participants performed better in terms of accuracy compared to tRNS, while no differences were found in the RTs. This result suggests that tRNS could be of no benefit to individuals who have already reached a high level of performance. 

In sum, 8 out of 12 tDCS studies found a beneficial effect as well as 3 out of 4 tRNS studies. Figure 4 (panel C) summarizes and compares the effects of tDCS and tRNS in arithmetic processing. The effects of tACS were also not considered since only one study was found, although with positive results.

Box 1 presents the overview of the main findings of the systematic review.

Box 1 **Overview of the main findings.**

1. The most effective tES set-up:
  → *Non-symbolic and symbolic number processes:*
  - Anodal tDCS over (left or right) parietal regions;
  - Bilateral tDCS over frontal regions;
  - Bilateral tRNS over parietal and frontal regions.
  → *Arithmetic processes:*
  - Bilateral tDCS over parietal regions (addition problems);
  - Anodal tDCS over parietal regions (subtraction and multiplication problems);
  - Bilateral and left anodal tDCS over frontal regions;
  - Bilateral tRNS over parietal and frontal regions.
2. Short-, medium-, and long-term effects after tES interventions.
3. Little evidence of transfer effect (specificity of tES on trained materials).
4. tES is more effective during challenging and demanding conditions.
5. tDCS effect varies by hemispheric lateralization of the engaged process, individual traits, performance at baseline.

## 4. Discussion

The originality and novelty of our work relies in its efforts to find a common thread and an integrative view to translate such results into clinical or neuroenhancement applications. 

In the following, we will critically consider the results concerning non-symbolic and symbolic number processes and arithmetic processes. Afterwards, considerations of stimulation protocols for clinical translation as well as future directions and limitations will be discussed.

### 4.1. Does tES Consistently Enhance Numerical Cognition?

Despite plenty of published studies, the application of tES in numerical cognition is still a relatively novel field of research, considering that the oldest study was published in 2010. Its emergent phase justifies the heterogeneity found in the proposed methodology (i.e., stimulation protocols, tasks, or training employed) and the absence of clinical trials for neurodevelopmental disorders related to numerical cognition, such as dyscalculia. 

Overall, this systematic review reveals that most studies (20 out of 26) showed the effectiveness of tES in improving certain aspects of numerical cognition, whereas only a small number of studies documented tES null or worsened effects.

### 4.2. What Are the Numerical Cognition Aspects in Which tES Would Be More Effective? What tES Technique Would Be More Effective in Ameliorating Certain Numerical Cognition Aspects and under Which Stimulated Brain Regions?

Overall, the majority of studies reported beneficial tES effects on non-symbolic and symbolic number processes as well as arithmetic processes (respectively, 80% vs. 76%). Our findings suggest that tES can improve both aspects of numerical cognition, with a tendency to be more effective at affecting number processes.

Given only a study that applied tACS on non-symbolic and symbolic number processes [75], we discussed and compared results across tDCS and tRNS studies. 

All tRNS studies reported positive effects (two studies) while all but one tDCS studies produced beneficial findings. 

In particular, when comparing tDCS and tRNS over parietal regions, tES reported overall positive results on number processing (four tDCS studies [70,71,73,76]; one HD-tDCS study [74]; two tRNS studies [77,78]). It should be noted that given the variabilities and heterogeneities in tES and task/training parameters, a direct comparison of studies was difficult, and results should be interpreted with caution. However, two tentative considerations may be relevant. 

First, the benefits of a *bilateral* parietal tDCS on number processing seem to be inconsistent, likely depending on the polarity of the montage and on the process intended to enhance (e.g., learning vs. automaticity). Concerning polarity, only a bipolar montage with cathodal/anodal tDCS over left/right PPC determined a more consistent improvement in some aspects of number processing compared to the reverse montage (anodal/cathodal over left/right PPC) and the placebo condition [70]. Concerning the target process, the unsuccessful montage of the aforementioned study (anodal/cathodal over left/right PPC) was found to enhance numerical learning—the acquisition process of numerical information—with a detrimental effect on numerical automaticity performance—the ability to effortlessly process numerical information [76]. Second, parietal anodal tDCS (regardless of lateralization; right tDCS [73], right or left tDCS [74]) and tRNS [77,78] are more likely to obtain improvements in terms of basic numerical processes. 

When comparing tDCS and tRNS to dlPFC, the application of tES determined positive results in two studies [70,79]; only anodal stimulation over right dlPFC produced a null effect [73]. 

Once again, the mixed methodology of the reviewed studies does not facilitate the extraction of the key findings. However, it could be carefully noted that the benefits of prefrontal stimulation on number processing seem to depend on the process intended to enhance. The extracted evidence showed that anodal/cathodal tDCS over left/right dlPFC enhanced numerical automaticity with even a detrimental effect on numerical learning [76]. Moreover, only sparse evidence of a lateralization-dependent effect was documented with no effect after excitatory stimulation of the dlPFC [73]. 

To summarize, both tDCS (regardless of polarity/lateralization) and tRNS (even with only two studies existing) over dlPFC would be advantageous to improve number processing. 

All but one tRNS study reported positive effects on arithmetic processes (3 out of 4 studies) while tDCS produced 67% of beneficial findings (8 out of 12 studies).

In particular, when comparing tDCS and tRNS over parietal regions, the application of tES induced contrasting results in terms of arithmetic process improvement. 

Specifically, only a bilateral tDCS montage over parietal regions would enhance *addition calculation* [82,91]. In contrast, the unipolar anodal or cathodal tDCS [85] and tRNS [89] did not affect addition performance. 

Moreover, after applying anodal tDCS over the right hemisphere or anodal/cathodal tDCS over left/right PPC, two studies (respectively, [80,87]) did not find the effects on *subtractions* and *simple multiplication problems*. Whereas, after anodal tDCS over left PPC areas, positive effects on mental calculations were obtained (i.e., complex subtraction problems [93]; double-digit subtraction tasks [71]; multiplication problems [81]), especially when the difficulty of the calculation problems increased [86]. Similarly, the only tRNS study over bilateral PPC [88] and the one study with a mixed montage (3 days of tRNS over bilateral dlPFC plus 2 days of tRNS over bilateral PPC [94]) showed positive results on mental calculations regardless of the type (multiplications, additions, and subtractions). 

The inconsistency of these results could be explained by the high variability of lateralization across individuals during arithmetic tasks. As the study by Kasahara et al. [81] underlined, the inter-individual variability in functional lateralization across individuals is very high for arithmetic processes, and this variability could contribute to significantly affecting tDCS results. Moreover, another explanation for these mixed results could be the indistinct inclusion of different types of calculations (such as addition, subtraction, and multiplication problems) during stimulations of parietal regions without considering the influence of the tDCS montage polarity. For instance, the study by Hauser et al. [87] assessed both complex subtractions and simple multiplication problems (i.e., arithmetic facts) as the main numerical cognition outcomes during and after participants receiving anodal/cathodal tDCS over left/right PPC. The authors failed to find some stimulation effects. This null evidence could be explained by some findings showing that, during subtraction and multiplication, left and right parietal regions are differently recruited [6]. Specifically, brain activity seems to be dominant in the bilateral or left hemisphere for subtractions and primarily in the right hemisphere for multiplications [6]. Therefore, when applying tDCS over parietal regions during or after arithmetic tasks, it is important to be particularly cautious to the montage and/or the polarity in light of the lateralization associated with the arithmetic tasks and of the high individual variability. 

When comparing tDCS and tRNS over dlPFC, studies found that tES significantly enhanced arithmetic processes [83,88,89,91,92] especially when the task was demanding [84]. Specifically, both bilateral tDCS and tRNS over dlPFC led to consistent improvements [83,88,91,92]. Similarly, anodal tDCS over left dlPFC was effective in enhancing large subtractions [91]. The only exception was the null result obtained by Krause et al. [90], probably because tRNS was proposed to participants who already reached the highest levels of performance (to mathematically highly-proficient, healthy, postgraduate students). 

Despite methodological heterogeneity across studies, we should note that polarity-independent tES, such as tRNS, would more likely result in enhancing certain aspects of arithmetic processes regardless of target brain regions. 

### 4.3. Considerations for Clinical Translation and Future Research 

Research on tES effects in healthy participants could lead to performance improvements, reducing time and effort. However, tES, due to its effectiveness, ease of use, and feasibility, could also provide highly beneficial impacts in children with neurodevelopmental disorders. For these reasons, increasing the number of studies that apply such brain-directed techniques in the clinical populations, especially for those is which evidence-based treatment is lacking, should be recommended. 

Of note, all but one study [69] recruited healthy adults or elderly individuals with a subsequent scarce presence of works in the clinical population, especially those with dyscalculia. Dyscalculia is a neurodevelopmental disorder that refers to a pattern of difficulties characterized by problems in processing numerical information, learning arithmetic facts, and performing accurate or fluent calculations [96]. With a prevalence of 5% to 6% [16], dyscalculia negatively affects academic and professional careers as well as emotional development. However, to date, standardized evidence-based interventions for dyscalculia are still not available. 

Looi et al. [69] conducted the first tRNS pilot study in children with dyscalculia. The between-subjects, pseudorandomized, single-blind study explored the effects of four sessions of tRNS combined with a *number line training* for 10 days over bilateral dlPFC in twelve children with dyscalculia. Results showed that the tRNS increased more of a number line accuracy (as a function of days of training) than sham tRNS. Moreover, the percentage of accuracy change in the number line was positively and highly correlated with the change in standardized mathematical tests.

Future research should propose randomized clinical trials to evaluate the effectiveness of tES in children and adults with dyscalculia. 

Despite the heterogeneity across the reviewed studies, some considerations for accelerating the translation of such tES findings into clinical settings as cognitive enhancement or brain-based treatments could be proposed.

1. Optimizing tES protocols. Further studies are needed to clarify the optimal tES parameters and procedures to alleviate or enhance some aspects of numerical cognition. However, taking our results together, we suggest that anodal tDCS (regardless of lateralization) over parietal regions, bilateral tDCS (regardless of polarity/lateralization) over frontal regions, and tRNS (regardless of brain regions) should be further investigated and validated as promising brain stimulation protocols to consistently improve *non-symbolic and symbolic number processes*. Within arithmetic processes, we suggest that bilateral tDCS over parietal regions be explored to specifically enhance *addition calculation* as well as left anodal tDCS and tRNS over parietal regions for *subtraction* and/or *multiplication problems*. Left anodal or bilateral tDCS and tRNS over frontal regions should be further investigated as effective protocols to enhance calculation skills, under demanding conditions. These considerations allow one to personalize and focus tES intervention on the specific individual weaknesses of numerical cognition areas, such as the number or arithmetic processes. It augments the potentiality for an individualized treatment. However, our findings show that tACS improves arithmetic learning faster than tDCS [95]. Further research with cutting-edge tES techniques, such as tACS, should be applied in the field to provide more focal, brain-tuned, and personalized brain stimulation and hopefully reduce the variability of the findings.
2. Investigating long-term improvements following tES interventions. Overall, only a few studies included follow-up assessments after a time window from the end of the tES intervention. Interestingly, in all of these studies, the beneficial effects appeared to be short-, medium- or long-lived. Of importance, it seems that only a few sessions of tES (e.g., from two to six) produced robust improvements that persisted even at the 2-month follow-up [79], at the 4-month follow-up [77,78] and the 6-month follow-up [70,92], regardless of the target processes (numerical or arithmetic) and tES technique (tDCS or tRNS). However, it is noteworthy to note that in all of these studies, tES was delivered *online*, together with a concomitant activity (i.e., cognitive training). Converging evidence suggests that tES has the potential to prompt training or task-induced neuroplasticity, facilitating brain activity underlying the engaged cerebral network [97,98]. The persistent beneficial outcomes could be due to the potential synergy between tES and concomitant training or tasks [13,97]. If we consider that short and intensive interventions (including only a handful of tES sessions combined with cognitive training) turn out to have long-term beneficial consequences, it becomes clear that the potential for translating tES interventions in clinical settings would be high and advantageous for patients, clinicians, and the healthcare system in terms of compliance, money, time, and resources. We, therefore, recommend implementing brain stimulation treatments combined with concomitant training, and assessing long-term effects following tES interventions, stand-alone and (especially) when combined with cognitive training, to validate their long-lasting effects on numerical cognition processes.
3. Evaluating the transfer effects. Another variable that would advance the application of tES in clinical settings is the transferability of training-related performance gains achieved during stimulation. What most people would expect of a tES intervention combined with cognitive training is improvements in their training-related cognitive abilities, useful in other contexts or other tasks, not just a better performance specific to the trained task. The transfer effect of tES combined with cognitive training remains a major criticism. Out of the eight studies assessing transfer effects of tES plus cognitive training, only four support the transferability of training-related performance improvement during stimulation to untrained skills [88,92,93,94]. The basic theory behind the *transfer* explains that if a brain network that is activated during cognitive training overlaps with networks related to untrained training/tasks, these neural networks will also be reinforced following the Hebbian learning rule and, consequently, produce enhanced cognitive performance on the untrained training/tasks [99]. A possible hypothesis for the unsuccessful transfer could be the insufficient practice on the trained tasks to generalize competencies or the fact that the training was not strong enough to induce the cortical changes that facilitate the generalization of learned skills or the use of inappropriate untrained skills/tasks [100]. Future research should focus on the optimal factors in terms of the number of sessions, reliability of the training, tES parameters that induce the transferability, and the maintaining of the training-related improvement achieved during stimulation.
4. Exploring neurobiological and neurophysiological tES effects. Investigating the neurobiological changes during or after tES intervention could clarify mechanisms underlying the behavioral changes and null or unclear results. While in some studies behavioral effects were found, in others, neurophysiological changes were detected without clear behavioral effects (i.e., Hauser et al. [87]). It seems that, although not immediately apparent from behavioral data, neurophysiological changes could be generated anyway and lead to long-term behavioral changes. There is a primary need to clarify whether neurophysiological effects appear immediately after a single session or emerge after multiple sessions of tES and their relation to the behavioral changes. Moreover, the functional reorganization of neural networks, along with the improvements of numerical and arithmetical abilities following tES interventions, are still open points in the literature.
5. Considering individual variability. It is well-recognized that the effects exerted by tES critically depend on the individual pre-conditions and online brain activity [101]. Accordingly, inter-individual variability of baseline performance and neurophysiological state could have affected tES impact on numerical cognition. Consistently, two reviewed studies showed that tES outcomes depend on individual traits, such as mathematical anxiety [83] and performance at baseline as mental calculation abilities [90].
6. Considering cognitive costs. The study by Iuculano and Cohen Kadosh [76] demonstrated that applying tES during numerical learning can lead to *cognitive enhancement* but also *cognitive impairment*. Namely, tDCS over bilateral dlPFC improved the automaticity of learned material but negatively affected the learning rate. Conversely, tDCS over bilateral PPC enhanced the learning rate but worsened automaticity. These findings suggest that the inclusion of diverse tasks could help to better understand the possible cost(s) and limitations of tES on cognitive processes. Researchers should then develop optimal stimulation parameters for cognitive enhancement, to better consider the cognitive costs of tES.

### 4.4. Limitations 

Some limitations of the reviewed studies should be underlined. They could have significantly affected the results of the studies as well as their interpretations in the current review. 

The common limits to all studies are especially related to the absence of a randomization method description (e.g., computer-generated random numbers, reference to a random number table, etc.) and the absence of *a priori* sample size calculations. 

The former limitation may be problematic for the replication of experimental methods and the main findings. The latter would turn out to be problematic for the robustness of results, considering the possibility of unpowered studies. This may produce bias and unexpected results. It should therefore be recommended, calculating the number of participants, *a priori*, using software, such as G*Power the G*Power Team, Düsseldorf, Germany), ensuring the soundness of the research. During *a priori* sample size calculations, the power should be set at >0.80 (ideally 0.95). The expected effect size (e.g., partial eta squared or Cohen’s f) on a specific task should rely on the previous studies with comparable outcomes, and researchers should take a conservative approach by estimating for somewhat smaller effect sizes than reported in the literature [102]. 

Other limitations may be encountered in the use of a single-blind instead of a double-blind design and in the lack of information regarding differences at baseline between experimental and placebo groups. Indeed, the placebo control group should be carefully matched for baseline performance as well as for confounding variables (i.e., age, gender, age of formal education, etc.) known to be relevant for the targeted functions and for tES effectiveness. 

Of importance, half of studies did not report effect sizes along with inferential statistics and significance nor individual data. Therefore, the final considerations of the current review rely only on the results interpreted through statistical significance. However, reporting the effect sizes along with the inferential statistics is fundamental because it helps readers understand the magnitude of the differences found, not just the possibility of a difference occurring. Therefore, it could be suggested to report both *p*-value and effect sizes, even when a significant difference does not emerge. 

Another factor that may have influenced the comprehensive findings of the current review could be identified in the heterogeneity of study designs (between vs. within-subjects). On the one hand, a within-subjects design might have reduced the influence of individual characteristics. However, it may have influenced participants’ beliefs about specific stimulation conditions, leading to ineffective blinding. Conversely, a between-subjects design would have reduced the risk of ineffective blinding but would have increased subject-specific variance. 

In conclusion, more standardized tES protocol studies on numerical cognition should be published and studies should be pre-registered—for two main reasons. The first one is to overcome publication bias. Publication bias refers to a systematic preferential publication of studies with significant positive or negative results; thus, excluding studies with null results. Over time, this practice may cause a distortion in the overview of the scientific literature and slow down the advancement of scientific knowledge. This may be especially problematic in light of the general replicability crisis in studies of cognitive neuroscience and non-invasive brain stimulation studies [103]. The second one is to reduce the diversity of the implement methodology across the studies. Publishing study protocols and explaining the key methodological aspects of tES interventions could increase scientific production, strengthen international research towards a common goal and, therefore, accelerate its clinical application [104]. Moreover, the inclusion of additional information to supplement the original research article (as research data, experimental tasks, analysis scripts) to ensure a better replication of the results is required. 

## 5. Conclusions

Although tES on numerical cognition has been increasingly used in the past two decades, heterogeneity in the methodology employed still makes it difficult to apply clinically. 

In this systematic review, we endeavored to find a bridge between experimental findings and clinical implementations.

Most of the reviewed studies applied tDCS and tRNS, while a few research studies used focal and brain-tuned techniques, such as HD-tDCS or tACS. We extrapolated and discussed specific electrodes placement, polarization, hemispheric lateralization, and brain regions, and their impacts in improving number and arithmetic processes.

In brief, anodal tDCS over parietal regions and bilateral tDCS over frontal regions strongly enhance number processing; bilateral tDCS over parietal and frontal regions and left anodal tDCS over frontal regions improve arithmetic skills; bilateral tRNS regardless of brain regions (parietal or frontal) is effective at improving number and arithmetic processes. 

Our findings show that tRNS, thus far, seems to be the most promising tES technique to enhance numerical cognition. 

Although the application of tES as a neuroenhancement or treatment approach in numerical cognition is promising, some questions remain. The optimal parameters and procedures of tES, the transfer effect to untrained numerical aspects, the sustained improvements in different aspects of numerical cognition, and the benefits of applying advanced tES techniques (i.e., HD-tDCS, tACS) have yet to be determined.

We believe that our review will provide insight when translating our findings into a clinical trial. 

## Figures and Tables

**Table 1:** Summary of results for non-symbolic and symbolic number processes and characteristics of the reviewed tES studies, according to the population, intervention, comparison, outcomes, and study design (PICOS) approach. Studies are presented in order of citation in the text, Section 3.3. Legend: a.r. = age range, M/F = male/females, y = years, NR = not reported, Exp. = experiment, tDCS = transcranial direct current stimulation, HD-tDCS = high-definition transcranial direct current stimulation, tRNS = transcranial random noise stimulation, tACS = transcranial alternating current stimulation, mA = milliampere, Hz = hertz, Ref = Reference, A = anodal, C = cathodal, cm^2^ = square centimeters, min = minutes, s = seconds, cSO = contralateral supraorbital, PPC = posterior parietal cortex, dlPFC = dorsolateral prefrontal cortex, RTs = reaction times, wf = Weber fraction.

First Author, Year, Country	Population Characteristics	Study Design	tES Protocol										Outcomes	Results
			tES	Current (Frequency)	Target Electrodes Size	Ref Electrodes Size	Duration	Session(s)	Brain Target	Ref Target	Montage and Conditions	Timing		
Cohen Kadosh et al., 2010 United Kingdom	A total of 15 healthy adults, a.r. 20–22 y, M/F NR, right-handed	Between-subjects, randomized, single-blind	tDCS	1 mA	9 cm^2^	-	20 min	6	PPC	-	Left anodal P3/Right cathodal P4 + training Left cathodal P3/Right anodal P4 + training Sham + training	Offline	Numerical Stroop task with artificial digits (RTs) Number line task with artificial digits (accuracy)	Left cathodal/right anodal tDCS improved both outcome measures
												Offline	Numerical Stroop task with everyday digits (RTs) Number line task with everyday digits (accuracy)	No effects ^a^
												Offline ^b^	Numerical Stroop task with artificial digits (RTs) Number line task with artificial digits (accuracy)	Left cathodal/ right anodal tDCS maintained the effects
Iuculano and Cohen Kadosh, 2013 United Kingdom	A total of 19 healthy adults, a.r. 20–31 y, 10 M/9 F, handedness NR	Between-subjects, randomized, single-blind	tDCS	1 mA	9 cm^2^	-	20 min	6	PPC dlPFC	-	Left anodal P3/Right cathodal P4 Left anodal F3/Right cathodal F4 Sham	Online	Artificial symbols training (RTs)	Left anodal/right cathodal over PPC improved performance
												Offline	Numerical Stroop task with artificial digits (RTs)	Left anodal/right cathodal over dlPFC improved performance
												Offline	Numerical Stroop task with everyday digits (RTs)	No effects ^a^
Hauser et al., 2013 Switzerland	Exp 1: 16 healthy adults, 22.8 ± 3.1 y, 10 M/11 F, right-handed	Within-subjects, counterbalanced, single-blind	tDCS	1 mA	35 cm^2^	100 cm^2^	20 min	1	PPC	cSO/ right eyebrow	Anodal P3/P4 Cathodal P3/P4 Left anodal P3 Left cathodal P3 Sham	Offline	Double-digit number comparison task Double-digit subtraction task (accuracy, RTs)	Left anodal tDCS improved both outcome measures
	Exp 2: 16 healthy adults, 23.6 ± 2.4 y, 7/9, right-handed	Within-subjects, counterbalanced, single-blind	tDCS	1 mA	35 cm^2^	100 cm^2^	20 min	1	PPC	cSO	Right anodal P4 Sham	Offline	Double-digit number comparison task Double-digit subtraction task (accuracy, RTs)	No effects
Li et al., 2015 Japan	A total of 18 healthy adults, a.r. 20–42 y, 9 M/9 F, right-handed	Within-subjects, counterbalanced, single-blind	tDCS	1 mA	35 cm^2^	-	30 min	1	PPC	-	Left anodal P3/Right cathodal P4 Left cathodal P3/Right anodal P4 Sham	Online	Number comparison task (RTs)	Left cathodal/right anodal tDCS worsened the performance
Brezis et al., 2016 Israel	Exp. 3: 12 healthy adults, age NR, M/F NR, right-handed	Within-subjects, counterbalanced, single-blind	tDCS	1 mA	9 cm^2^	15 cm^2^	25 min	1	PPC dlPFC	cSO	Right anodal P4 Right anodal F4 Sham	Online	Numerical averaging task (accuracy)	Right anodal tDCS over PPC improved performance
Looi et al., 2016 United Kingdom	A total of 30 healthy adults, 24.2 ± 2.10 y, 10 M/20 F, right-handed	Between-subjects, randomized, single-blind	tDCS	1 mA	35 cm^2^	-	30 min	2	dlPFC	-	Left cathodal F3/Right anodal F4 Sham	Online	Number line training (accuracy, RTs)	Left cathodal/right anodal tDCS improved performance
												Offline ^c^	Number line training (accuracy, RTs)	Left cathodal/right anodal tDCS maintained the effects
Hartmann et al., 2020 Switzerland	A total of 18 healthy adults, a.r. 22–30 y, 10 M/8 F, handedness NR	Within-subjects, counterbalanced, single-blind	HD-tDCS	A: 2 mA; C: −0.5 mA	0.79 cm^2^	-	25 min	1	PPC	-	Left anodal P3 Right anodal P4 Sham	Online	Non-symbolic approximate arithmetic task (accuracy)	Right anodal tDCS improved performance
Cappelletti et al., 2013 United Kingdom	A total of 40 healthy adults, a.r. 19–36 y, 18 M/22 F, right-handed	Between-subjects, randomized, double-blind	tRNS	±1 mA (0–250 Hz)	35 cm^2^	-	20 min	5	PPC Motor areas	-	P3/P4 + training C3/C4 + training P3/P4 Sham + training	Online/ Offline	Numerosity discrimination training (*wf*)	tRNS over PPC improved performance
												Offline	Numerical Stroop task Non-symbolic approximate arithmetic task Arithmetical processing task (accuracy, RTs)	No effects ^a^
												Offline ^d^	Numerosity discrimination training (*wf*)	tRNS over PPC improved performance
Cappelletti et al., 2015 United Kingdom	A total of 60 healthy adults, a.r. 19–73 y, 25 M/35 F, right-handed	Between-subjects, randomization NR, double-blind	tRNS	±1 mA (0.1–640 Hz)	35 cm^2^	-	20 min	5	PPC Motor areas	-	P3/P4 + training C3/C4 + training Sham + training	Online/ Offline	Numerosity discrimination training (*wf*)	tRNS over PPC improved performance
												Offline	Numerical Stroop task Non-symbolic approximate arithmetic task Arithmetical processing task (accuracy, RTs)	No effects ^a^
												Offline ^d^	Numerosity discrimination training (*wf*)	tRNS over PPC improved performance
													Numerical Stroop task Non-symbolic approximate arithmetic task Arithmetical processing task (accuracy, RTs)	No effects
Labree et al., 2020 United Kingdom	Exp 1: 31 healthy adults, a.r. 18–34 y, 9 M/22 F, right-handed	Within-subjects, counterbalanced, double-blind	tACS	±1.5 mA (in-phase 0°)	35 cm^2^	-	10 min (fade in/out period of 20 s)	1	PPC	-	Theta-tACS P3/P4 Alpha-tACS P3/P4 Beta-tACS P3/P4 Sham	Online	Numerosity discrimination task (*wf*)	Alpha-tACS over PPC specifically worsened performance
	Exp 2: 25 healthy adults, a.r. 18–37 y, 4 M/21 F, right-handed	Within-subjects, counterbalanced, double-blind	tACS	±1.5 mA (in-phase 0°)	35 cm^2^	-	10 min (fade in/out period of 20 s)	1	PPC dlPFC	-	Alpha-tACS P3/P4 or F3/F4 Sham	Online	Numerosity discrimination task (*wf*)	Alpha-tACS over PPC specifically worsened performance

^a^ Transfer effects; ^b^ 6-month follow-up; ^c^ 2-month follow-up; ^d^ 4-month follow-up.

**Table 2:** Summary of results for arithmetic processes and characteristics of the reviewed tES studies according to the population, intervention, comparison, outcomes and study design (PICOS) approach. Studies are presented in order of citation in the text, Section 3.4. Legend: a.r. = age range, M/F = male/females, y = years, NR = not reported, Exp. = experiment, tDCS = transcranial direct current stimulation, tRNS = transcranial random noise stimulation, tACS = transcranial alternating current stimulation, mA = milliampere, Hz = hertz, Ref = Reference, A = anodal, C = cathodal, cm^2^ = square centimeters, min = minutes, s = seconds, cSO = contralateral supraorbital, PPC = posterior parietal cortex, dlPFC = dorsolateral prefrontal cortex, RTs = reaction times.

First Author, Year, Country	Population Characteristics	Study Design	tES Protocol										Outcomes	Results
			tES	Current (Frequency)	Target Electrodes Size	Ref Electrodes Size	Duration	Session(s)	Brain Target	Ref Target	Montage and Conditions	Timing		
Clemens et al., 2013 Germany	A total of 10 healthy adults, 43 ± 12.4 y, 10 M/0 F, right-handed	Within-subjects, counterbalanced, single-blind	tDCS	2 mA	35 cm^2^	35 cm^2^	20 min	1	PPC	cSO	Right anodal CP4 Sham	Offline	Simple multiplications verification task (efficiency)	No effects
Klein et al., 2013 Germany	A total of 24 healthy adults, a.r. 20–44 y, 10 M/14 F, 23 right-handed and 1 left-handed	Within-subjects, counterbalanced, blinding NR	tDCS	1 mA	35 cm^2^	100 cm^2^	20 min	1	PPC	cSO	Anodal P3/P4 Cathodal P3/P4 Sham	Online	Addition task (RTs)	Bilateral anodal tDCS improved performance
Kasahara et al., 2013 Japan	A total of 16 healthy adults, a.r. 20–23 y, 11 M/5 F, right-handed	Crossover design (2 groups: LPHD group *vs* BPHD group), randomization NR, single-blind	tDCS	2 mA	35 cm^2^	-	10 min	1	PPC	-	Left anodal P3/ Right cathodal P4 Left anodal P3/ Right cathodal P4 Sham	Online	Mental calculation task (RTs)	Only in LPHD group, Left anodal/right cathodal tDCS improved performance
												Offline	Mental calculation task (RTs)	No effects
Sarkar et al., 2014 United Kingdom	A total of 45 healthy adults, 22.47 ± 3.31 y, 23 M/22 F, left-handed	Crossover design (2 groups: HMAnx Group vs. LMAnx Group), randomized, double blind	tDCS	1 mA	25 cm^2^	-	30 min	2	dlPFC	-	Left anodal F3/Right cathodal F4 Sham	Online	Simple arithmetic decision task (RTs)	In HMAnx Group, Left anodal/right cathodal tDCS improved performance
Grabner et al., 2015 Switzerland	A total of 60 healthy adults, 21.98 ± 2.99 y, 30 M/30 F, right-handed	Between-subjects, randomized, double-blind	tDCS	1.5 mA	35 cm^2^	100 cm^2^	30 min	1	PPC	cSO	Left anodal P5-CP5 Left cathodal P5-CP5 Sham	Online	Complex multiplications and subtractions (accuracy, RTs)	Left anodal tDCS improved accuracy in subtractions; left cathodal tDCS increased RTs in both tasks
												Offline ^b^	Complex multiplications subtractions (trained and untrained problems, accuracy, RTs)	The negative effects of left cathodal tDCS were maintained only in trained problems ^a^
Rütsche et al., 2015 Switzerland	A total of 23 healthy adults, 21.8 ± 2.66 y, 6 M/17 F, right-handed	Within-subjects, randomized, single-blind	tDCS	1.5 mA	35 cm^2^	100 cm^2^	30 min	1	PPC	cSO	Left anodal P5-CP5 Sham	Online	Additions and subtractions (small vs. large, RTs)	Left anodal tDCS improved performance
Pope et al., 2015 United Kingdom	A total of 59 healthy adults, 21.8 ± 3.7 y, 18 M/41 F, handedness NR	Between-subjects, randomized, single-blind	tDCS	2 mA	25 cm^2^	25 cm^2^	20 min	1	dlPFC	deltoid muscle	Left anodal F3 Left cathodal F3 Sham	Offline	PASAT/ PASST (accuracy, RTs)	Left anodal tDCS improved performance in the PASST
Artemenko et al., 2015 Germany	A total of 25 healthy adults 23.28 ± 4.51 y, 3 M/22 F, right-handed	Within-subjects, counterbalanced, blinding NR	tDCS	1 mA	35 cm^2^	100 cm^2^	20 min	1	PPC	cSO	Left cathodal P3 Left anodal P3 Right cathodal P4 Right anodal P4 Sham	Online	Addition task (RTs)	No effects
Hauser et al., 2016 Switzerland	A total of 40 healthy adults, 22.40 ± 3.3 y, 20 M/20 F, right-handed	Between-subjects, randomized, double-blind	tDCS	1 mA	35 cm^2^	50 cm^2^	30 min	1	PPC	Fpz-AF8	Left anodal P5-CP5 Sham	Online	Complex subtractions (arithmetic facts retrieval, calculations; accuracy, RTs)	No effects
												Offline	Complex multiplications subtractions (trained and untrained problems; accuracy, RTs)	No effects
Mosbacher et al., 2020 Austria	A total of 62 healthy adults, 25.9 ± 5.1 y, 24 M/38 F, right-handed	Between-subjects, randomized, double-blind	tDCS	1 mA	9 cm^2^	35 cm^2^	25 min	1	PPC dlPFC	cSO	Left anodal P3 or F3 Sham	Online	Additions and subtractions (small vs. large; RTs)	Left anodal tDCS over dlPFC improved performance only in the large subtractions
												Offline	Additions and subtractions (small vs. large; RTs)	Left anodal tDCS over dlPFC improved performance only in the large subtractions
Mosbacher et al., 2021 Austria	A total of 137 healthy adults, 22.5 ± 3.8 y, 48 M/89 F, right-handed	Between-subjects, randomized, double-blind	tDCS	1 mA	9 cm^2^	35 cm^2^	25 min	1	PPC dlPFC	cSO	Left anodal P3 or F3 Sham	Online	Arithmetic learning training (RTs)	No effects
												Offline	Arithmetic learning training (RTs)	No effects
			tACS	1–1.5 mA (100 periods of fade in/out phase)	9 cm^2^	35 cm^2^	25 min	1	PPC dlPFC	shoulder	Alpha-tACS P3 or F3 Theta-tACS P3 or F3 Sham	Online	Arithmetic learning training (RTs)	Theta-tACS over dlPFC reduced the repetitions needed to learn novel facts
												Offline	Arithmetic learning training (RTs)	Theta-tACS over dlPFC and PPC improved performance
Snowball et al., 2013 United Kingdom	A total of 25 healthy adults, 21.17 ± 2.67 y, 12 M/13 F, right-handed	Between-subjects, randomized, double-blind	tRNS	1 mA (100–600 Hz)	25 cm^2^	-	20 min	5	dlPFC	-	F3/F4 Sham	Online	Calculation learning training; Drill learning training (accuracy, RTs)	Bilateral tRNS improved performance
												Offline ^c^	Calculation learning training; Drill learning training (accuracy, RTs)	The effect was maintained only for calculation RTs for trained and untrained problems ^a^
Popescu et al., 2016 United Kingdom	A total of 32 healthy adults, 22.38 ± 3.37 y, 18 M/14 F, right-handed	Between-subjects, randomized, double-blind	tRNS	1 mA (100–640 Hz)	16 cm^2^	-	20 min	5	PPC dlPFC	-	P3/P4 + F3/F4 Sham	Online	Calculation learning training; Drill learning training (accuracy, RTs)	Bilateral tRNS improved performance
												Offline	Calculation learning training; Drill learning training (accuracy, RTs)	Bilateral tRNS improved performance ^a^
Pasqualotto, 2016 Turkey	A total of 54 healthy adults, 21.5 ± 3.37 y, 27 M/27 F, handedness NR	Between-subjects, randomized, double-blind	tRNS	1 mA (100–600 Hz)	25 cm^2^	-	20 min	1	PPC dlPFC	-	P3/P4 F3/F4 Sham	Online	Subtractions verification task (RTs)	Bilateral tRNS over PPC and dlPFC improved performance
												Offline ^d^	Subtractions verification task (trained + untrained; accuracy, RTs)	Bilateral tRNS over PPC and dlPFC improved performance in accuracy ^a^
Bieck et al., 2018 Germany	A total of 48 healthy adults 23.48 ± 3.30 y 19 M/29 F right-handed	Within-subjects, counterbalanced, single-blind	tRNS	±0.5 mA (100–640 Hz)	35 cm^2^	-	20 min	1	PPC dlPFC	-	P3/P4 F3/F4 Sham	Online	Addition task (RTs)	Bilateral tRNS over dlPFC produced a light improvement
Krause et al., 2019 United Kingdom	Exp. 2: 6 high proficient healthy adults, 28 ± 4.47 y, handedness NR	Within-subjects, counterbalanced, double-blind	tRNS	1 mA (0.1–500 Hz)	25 cm^2^	-	20 min	1	dlPFC	-	F3/F4 Sham	Online	Complex calculations task (accuracy)	Bilateral tRNS negatively affected performance

^a^ Transfer effects; ^b^ 24 h after; ^c^ 6-month follow-up; ^d^ 7 days after.

## Data Availability

Not applicable.

## Supplementary Materials

The following supporting information can be downloaded at: https://www.mdpi.com/article/10.3390/jcm11082082/s1, Table S1. PRISMA Checklist. Box S1. Overview of the trainings (part A) and tasks (part B) employed in the reviewed tES studies to enhance nonsymbolic and symbolic number processes. Box S2. Overview of the trainings (part A) and tasks (part B) employed in the reviewed tES studies to enhance arithmetic processes.
