# Supplementary material for: Understanding the Effects of Transcranial Electrical Stimulation in Numerical Cognition: A Systematic Review for Clinical Translation
Source: J Clin Med. 2022 Apr 7;11(8):2082. doi: 10.3390/jcm11082082 (PMC9032363; doi:10.3390/jcm11082082)
Supplement: Supplementary file 1 [file jcm-11-02082-s001.zip › jcm-1626407-supplementary.pdf]

Supplementary materials

Table S1. PRISMA Checklist.

Section and Topic	Item #	Checklist item	Location where item is reported
**TITLE**			
Title	1	Identify the report as a systematic review.	Page 1
**ABSTRACT**			
Abstract	2	See the PRISMA 2020 for Abstracts checklist.	Page 1
**INTRODUCTION**			
Rationale	3	Describe the rationale for the review in the context of existing knowledge.	Pages 1-7
Objectives	4	Provide an explicit statement of the objective(s) or question(s) the review addresses.	Pages 2-3
**METHODS**			
Eligibility criteria	5	Specify the inclusion and exclusion criteria for the review and how studies were grouped for the syntheses.	Page 7
Information sources	6	Specify all databases, registers, websites, organisations, reference lists and other sources searched or consulted to identify studies. Specify the date when each source was last searched or consulted.	Pages 7-8
Search strategy	7	Present the full search strategies for all databases, registers and websites, including any filters and limits used.	Pages 7-8
Selection process	8	Specify the methods used to decide whether a study met the inclusion criteria of the review, including how many reviewers screened each record and each report retrieved, whether they worked independently, and if applicable, details of automation tools used in the process.	Pages 7-8
Data collection process	9	Specify the methods used to collect data from reports, including how many reviewers collected data from each report, whether they worked independently, any processes for obtaining or confirming data from study investigators, and if applicable, details of automation tools used in the process.	Pages 7-8
Data items	10a	List and define all outcomes for which data were sought. Specify whether all results that were compatible with each outcome domain in each study were sought (e.g. for all measures, time points, analyses), and if not, the methods used to decide which results to collect.	Pages 7-8
	10b	List and define all other variables for which data were sought (e.g. participant and intervention characteristics, funding sources). Describe any assumptions made about any missing or unclear information.	Pages 7-8
Study risk of bias assessment	11	Specify the methods used to assess risk of bias in the included studies, including details of the tool(s) used, how many reviewers assessed each study and whether they worked independently, and if applicable, details of automation tools used in the process.	n.a.
Effect measures	12	Specify for each outcome the effect measure(s) (e.g. risk ratio, mean difference) used in the synthesis or presentation of results.	n.a.
Synthesis methods	13a	Describe the processes used to decide which studies were eligible for each synthesis (e.g. tabulating the study intervention characteristics and comparing against the planned groups for each synthesis (item #5)).	Pages 7-8
	13b	Describe any methods required to prepare the data for presentation or synthesis, such as handling of missing summary statistics, or data conversions.	n.a.
	13c	Describe any methods used to tabulate or visually display results of individual studies and syntheses.	Pages 7-8

Section and Topic	Item #	Checklist item	Location where item is reported
	13d	Describe any methods used to synthesize results and provide a rationale for the choice(s). If meta-analysis was performed, describe the model(s), method(s) to identify the presence and extent of statistical heterogeneity, and software package(s) used.	n.a.
	13e	Describe any methods used to explore possible causes of heterogeneity among study results (e.g. subgroup analysis, meta-regression).	n.a.
	13f	Describe any sensitivity analyses conducted to assess robustness of the synthesized results.	n.a.
Reporting bias assessment	14	Describe any methods used to assess risk of bias due to missing results in a synthesis (arising from reporting biases).	n.a.
Certainty assessment	15	Describe any methods used to assess certainty (or confidence) in the body of evidence for an outcome.	n.a.
**RESULTS**			
Study selection	16a	Describe the results of the search and selection process, from the number of records identified in the search to the number of studies included in the review, ideally using a flow diagram.	Pages 8-25
	16b	Cite studies that might appear to meet the inclusion criteria, but which were excluded, and explain why they were excluded.	Pages 8-25
Study characteristics	17	Cite each included study and present its characteristics.	Pages 8-25
Risk of bias in studies	18	Present assessments of risk of bias for each included study.	n.a.
Results of individual studies	19	For all outcomes, present, for each study: (a) summary statistics for each group (where appropriate) and (b) an effect estimate and its precision (e.g. confidence/credible interval), ideally using structured tables or plots.	n.a.
Results of syntheses	20a	For each synthesis, briefly summarise the characteristics and risk of bias among contributing studies.	n.a.
	20b	Present results of all statistical syntheses conducted. If meta-analysis was done, present for each the summary estimate and its precision (e.g. confidence/credible interval) and measures of statistical heterogeneity. If comparing groups, describe the direction of the effect.	n.a.
	20c	Present results of all investigations of possible causes of heterogeneity among study results.	n.a.
	20d	Present results of all sensitivity analyses conducted to assess the robustness of the synthesized results.	n.a.
Reporting biases	21	Present assessments of risk of bias due to missing results (arising from reporting biases) for each synthesis assessed.	n.a.
Certainty of evidence	22	Present assessments of certainty (or confidence) in the body of evidence for each outcome assessed.	n.a.
**DISCUSSION**			
Discussion	23a	Provide a general interpretation of the results in the context of other evidence.	Pages 25-29
	23b	Discuss any limitations of the evidence included in the review.	Pages 29-30
	23c	Discuss any limitations of the review processes used.	Pages 29-30

Section and Topic	Item #	Checklist item	Location where item is reported
	23d	Discuss implications of the results for practice, policy, and future research.	Pages 25-29
**OTHER INFORMATION**			
Registration and protocol	24a	Provide registration information for the review, including register name and registration number, or state that the review was not registered.	Pages 1 and 7
	24b	Indicate where the review protocol can be accessed, or state that a protocol was not prepared.	Page 7
	24c	Describe and explain any amendments to information provided at registration or in the protocol.	n.a.
Support	25	Describe sources of financial or non-financial support for the review, and the role of the funders or sponsors in the review.	Page 31
Competing interests	26	Declare any competing interests of review authors.	Page 31
Availability of data, code and other materials	27	Report which of the following are publicly available and where they can be found: template data collection forms; data extracted from included studies; data used for all analyses; analytic code; any other materials used in the review.	n.a.

Liberati, A.; Altman, D.G.; Tetzlaff, J.; Mulrow, C.; Gøtzsche, P.C.; Ioannidis, J.P.; Clarke, M.; Devereaux, P.J.; Kleijnen, J.; Moher, D. The PRISMA statement for reporting systematic reviews and meta-analyses of studies that evaluate healthcare interventions: Explanation and elaboration. *BMJ* **2009**, *339*, b2700. https://doi.org/10.1136/bmj.b2700.

For more information, visit: http://www.prisma-statement.org/

Box S1. Overview of the trainings (part A) and tasks (part B) employed in the reviewed tES studies to enhance nonsymbolic and symbolic number processes.

Nonsymbolic and symbolic number processes

A) Trainings

Artificial symbols training

This training aimed at simulating numerical learning and creating a new number sense. Nine artificial meaningless symbols were arbitrarily assigned to the numbers 1-9. The training was on learning the implicit association between the nine arbitrary meaningless symbols and the corresponding quantity that had been assigned to them. Participants were not aware about the quantity correspondence. Each trial began with a fixation point at the centre of a black computer screen. When the fixation disappeared, two symbols appeared on the computer screen, one symbol was in the left visual field, and another one in the right visual field. Participants were instructed to choose the symbol they thought had a larger magnitude in each pair. Feedback was provided for every trial. For more details, see Cohen Kadosh et al. [1], and Iuculano and Cohen Kadosh [2].

Number line training

Participants were required to indicate the position of fractions by moving their body side-to-side on a virtual number line. The game was adaptive, with a range of difficulty levels depending on the performance. Participants received feedback at the end of each trial, and they were asked to respond as quickly and as accurately as possible. For more details, see Looi et al. [3].

Numerosity discrimination training

Participants were required to discriminate the numerosity of two sets of dots. Sets of intermixed, computer-generated dots were presented to participants who were instructed to respond indicating which set contained approximately more dots. Feedback was provided at the end of each trial. For more details, see Cappelletti et al. [4] and Cappelletti et al. [5].

B) Tasks

Numerical stroop task

a) Arabic digits. Participants were required to compare two numerical stimuli according to their physical size and to choose the physically larger digit by pressing a button as quickly and as accurately as possible. The stimuli could be incongruent (for example, a physically large 2 and a physically small 4), neutral (for example, a physically small 2 and a physically large 2), or congruent (for example, a physically small 2 and a physically large 4). No feedback was given. For more details, see Cohen Kadosh et al.[1], Iuculano and Cohen Kadosh [2], Cappelletti et al. [4], and Cappelletti et al. [5]

b) Artificial digits. In this task, the nine artificial meaningless symbols learned during the *Artificial symbols training* (see above) were employed instead of numbers. Like in the Arabic digits' version, participants were required to compare two artificial meaningless symbols according to their physical size and to choose the physically larger digit by pressing a button as quickly and as accurately as possible. The stimuli could be incongruent (i.e., the numerically larger digit was physically smaller), neutral (i.e., the digits differed only in the relevant dimension), or congruent (i.e., numerically larger digit was also physically larger). No feedback was given. For more details, see Cohen Kadosh et al. [1] and Iuculano and Cohen Kadosh [2].

Number line task

Artificial digits. In this task, the nine artificial meaningless symbols learned during the *Artificial symbols training* (see part A) were employed instead of numbers. Participants were asked to map artificial meaningless symbols onto a physical horizontal line. No feedback was given. For more details, see Cohen Kadosh et al. [1].

Number comparison task

a) Double digit. Participants were required to judge as fast as possible whether the presented Arabic number was bigger or smaller than 65. Presented Arabic numbers ranged between 31 and 99 (except 65), and each was presented twice. No feedback was given. For more details, see Hauser et al. [6].

b) Single digit. Participants were required to decide in a maximum of two seconds which was the bigger Arabic number between the two single-digit Arabic numbers presented (on the right and left). All possible Arabic single-digit numbers except 5 and 0 were used in all possible coupling, and presented randomly and twice during each block. No feedback was given. For more details, see Li et al. [7].

Numerical averaging task

Each trial began with a central fixation cross after which a sequence of two-digit numbers was presented (white Arabic numerals on black background). Participants were asked to convey as accurately as possible the sequence's average by vertically sliding a mouse-controlled bar set on a number ruler between 0 and 100 and to press the left mouse button when reaching the desired number. Each block terminated with performance feedback. For more details, see Brezis et al. [8].

Nonsymbolic approximate arithmetic task

Participants were instructed to approximately estimate as precisely and quickly as possible the outcome of an arithmetic addition or subtraction problem using dots. They were informed that they were not supposed to count the dots but rather intuitively select the solution that appeared to be most correct. A trial began with the information about the arithmetic operation. The word “addition” or “subtraction” appeared at the centre of the screen. After a short blank screen, the first operand appeared at the centre of the screen, immediately followed by the second operand. After another short blank, seven possible solutions appeared on the screen in a circular arrangement. After the onset of the solution screen, the mouse cursor appeared at the centre of the screen, and participants clicked at one of the seven proposed solutions. No feedback was provided. For more details, see Cappelletti et al. [4], Cappelletti et al. [5] and Hartmann et al. [9].

Numerosity discrimination task

Participants were presented with a series of intermixed yellow and blue dots of different sizes for a maximum time of 200 msec. They had to indicate by pressing a button whether each trial contained more blue or yellow dots. There was no time limit to respond. No feedback was given. For more details, see Labree et al. [10].

Box S2. Overview of the trainings (part A) and tasks (part B) employed in the reviewed tES studies to enhance arithmetic processes.

Arithmetic processes

A) Trainings

Calculation training

a) Complex multiplication and subtraction problems training. Participants were presented with the problems on a computer screen and were required to type in the answer as fast and as accurately as possible. The multiplication problems were two-digit per one-digit problems with two-digit solutions. The subtraction problems were two-digit minus two-digit problems with two-digit solutions. In the learning session, participants learned the solutions of complex multiplication and complex subtraction problems. Feedback was provided after each response. Twenty-four hours after the learning session, a performance session took place: participants were presented with old (trained) as well as new (untrained) multiplication and subtraction problems. No feedback was provided. For more details, see Grabner et al. [11].

b) Calculation learning training. Participants were instructed to answer to the presented calculation applying an algorithm. Answers to each calculation problem were obtained by manipulation of two numerical operands according to a particular algorithm (multiplication, addition, or subtraction). Participants were instructed to enter two-digit solution on the number pad of a keyboard. Positive and negative feedback was provided for each answer, and participants were allowed to progress to subsequent problems only once they reached the correct solution. For more details, see Snowball et al. [12] and Popescu et al. [13].

Drill learning training

Each trial began with the presentation of two numerical operands accompanied by the problem's answer. After the initial presentation, the problem disappeared from the screen and reappeared without the answer, at which point participants were required to enter their two-digit solution. Drill trials involved remembering associations between pairs of operands and a given result. The result of each drill problem was determined using a certain algorithm; participants were not given this algorithm and were instructed to not try and guess it, but instead to learn the associations purely by "drill". Positive and negative feedback was provided, and if participants answered incorrectly, the whole presentation cycle was repeated. For more details, see Snowball et al. [12] and Popescu et al. [13].

Arithmetic learning training

Participants were required to solve 196 arithmetic problems over the course of five blocks, spread over two different types of tasks (*Procedural learning trials* and *Fact learning trials*). Problems were presented as equations with unknowns (i.e. 3 – A*B + 3 = 5), and participants had to perform a series of basic arithmetic operations to determine the solution. In particular, problems in *Procedural learning trials* were never repeated and therefore always required procedural calculation, with the aim of training procedural calculation skills. Conversely, in *Fact learning trials* part of the trials were repeated identically throughout the blocks, requiring participants to acquire factual knowledge by memorising the solutions to the factual learning problems that were repeated during the task. Participants had a maximum of 8 s to solve the problem, and a further 3 s to choose from three solutions the correct one by pressing the respective button. After receiving feedback, they had to indicate which strategy they had used to solve the problem choosing between 3 options: (a) retrieved from memory, (b) calculated, and (c) neither. For more details, see Mosbacher et al. [14].

B) Tasks

Double-Digit subtraction task

Participants had to calculate the presented two-digit subtraction as fast as possible and indicate that they found the solution by pressing a button on the number pad of the keyboard. Subsequently, three result options were displayed, and the participants had to indicate the correct solution by pressing buttons on the number pad. Each time, 2 out of 6 distractors were randomly chosen and displayed together with the correct solution in a random order. No feedback was given. For more details, see Hauser et al. [6].

Arithmetical processing task

Participants were assessed on arithmetical tasks required to verify the result of addition, subtraction, and multiplication. The arithmetical problems included simple and complex exercises featured respectively by single and multi-digit operands. They were displayed with two one-digit or two-digit answers, one of which was correct. No feedback was given on the performance in these tasks. For more details, see Cappelletti et al. [4] and Cappelletti et al. [5].

Arithmetic verification tasks

a) *Multiplication verification task*. Simple multiplication problems with operands ranging from 0 to 10 were presented in Arabic format on a computer screen. Participants were instructed to decide as accurately and as fast as possible whether the presented solution of the multiplication problem was correct or incorrect. No feedback was given. For more details, see Clemens et al. [15].

b) *Subtraction verification task*. Participants were asked to judge the correctness of the subtraction results that appeared on a computer screen. Each subtraction was sequentially displayed. Participants were required to be as fast and as accurate as possible. Feedback was not provided. For more details, see Pasqualotto [16].

Mental calculation task
A multiplication problem comprising a two-digit number and a one-digit number was presented on the screen as “Problem”, followed by four three-digit numbers simultaneously presented under the prompt “Answer”. The participants were requested to multiply the two numbers presented in the “Problem”, and to choose the correct answer from the four options presented as quickly as possible by pushing a button within the response window. No feedback was given. For more details, see Kasahara et al. [17].

Simple arithmetic decision task
Participants were first primed with a valenced word (positive or negative), and then decided whether a simple equation was true or false. Every trial began with a fixation cross in the centre of the screen, followed by the presentation of the prime. The target equation was then presented, and the participant had to decide whether it was true or false by pressing buttons on the keyboard. No feedback was given. For more details, see Sarkar et al. [18].

Addition task
Participants were asked to solve two-digit addition problems by pressing a button in a maximum time of 5000 ms. Addition problems were presented together with two possible solutions, participants had to press the button corresponding to the correct one or closer to the correct one between the two options. No feedback was given. For more details, see Klein et al. [19], Artemenko et al. [20], and Bieck et al. [21].

Paced Auditory Serial Addition Task (PASAT) and Paced Auditory Serial Subtraction Task (PASST)
With regard to the PASAT, participants were required to listen to a series of numbers presented at either 2 or 3 s intervals, and to add the number they had just heard to the number immediately preceding the last one, answering aloud with the result. With regard to the PASST, the only difference with the PASAT is that participants did not have to add up but to subtract the number they had just heard from the number they heard before it. For both tasks, the numbers in the sequences were in the range of 1–9. No feedback was given. For more details, see Pope et al. [22].

Small vs large additions and subtractions
a) Participants were presented with addition and subtraction problems of small and large problem size. They were instructed to solve the problems as accurately and fast as possible and to speak the answer into a microphone as soon as they had found the solution. A single trial consisted of the presentation of a fixation point, followed by the arithmetic problem that disappeared as soon as an oral response was registered. Small problems were defined as one-digit/one-digit problems with addends between 2 and 8. Large problems were randomly selected from a pool of every possible two-digit/two-digit carry problem with addends ranging from 12 to 29. No feedback was provided. For more details, see Rütsche et al. [23].
b) Participants were asked to solve three sets of additions and three sets of subtractions. Each set consisted of easy problems to be solved by fact retrieval, and of large problems to be solved by procedural calculation. Easy problems were one-digit/one-digit problems (additions/subtractions) with a maximum sum of 10. Large problems were two-digit/two-digit problems (additions/subtractions) below 100. Problems were presented on screen until the participant pressed a button, indicating that the participant reached the solution. After the button was pressed, participants had to choose the correct solution from three options by pressing the corresponding button. After each set, participants had to indicate if they used fact retrieval or procedural strategies. No feedback was provided. For more details, see Mosbacher et al. [24].

Complex subtraction problems: arithmetic facts retrieval and calculations
Participants were presented with complex subtraction problems (e.g., two-digit minus two-digit problems). To administer two types of arithmetic demands, the subtraction problems were divided into two conditions. In the learning condition, 3 problems (randomly selected from a pool of problems) were presented several times over the course of the experiment (i.e., repeated problems). The high number of repetitions elicits fact learning, so that the participants could finally solve these problems by means of fact retrieval. To ensure that the participants learned the solutions to the repeated stimuli, the sessions were interspersed with short learning checks. In the no-learning condition, the remaining problems (i.e., novel problems) of the pool were presented only 3 times each to the participants, so that these problems needed to be solved by means of procedures rather than fact retrieval. In every trial, the arithmetic problem was presented, and then three choice alternatives were presented after a jittered fixation cross. The participants selected one of the alternatives. Feedback about the correctness of the response was provided. For more details, see Hauser et al. [25].

Complex calculation task
The problems presented on a computer screen involved either a 100- or a 120-digit number that had to be broken down into 20 different 6-digit prime number factors that, multiplied by each other in ascending order, made up the presented number. Participants had to either successfully identify one of these twenty prime numbers, or additionally provide the factor position within the multiplication sequence. The task provided feedback on the correctness of the response. For more details, see Krause et al. [26].

References

1. Cohen Kadosh, R.; Soskic, S.; Iuculano, T.; Kanai, R.; Walsh, V. Modulating Neuronal Activity Produces Specific and Long-Lasting Changes in Numerical Competence. *Current Biol.* **2010**, *20* (22), 2016–2020. https://doi.org/10.1016/j.cub.2010.10.007.

2. Iuculano, T.; Cohen Kadosh, R. The Mental Cost of Cognitive Enhancement. *J. Neurosci.* **2013**, *33* (10), 4482–4486. https://doi.org/10.1523/JNEUROSCI.4927-12.2013.

3. Looi, C. Y.; Duta, M.; Brem, A.-K.; Huber, S.; Nuerk, H.-C.; Cohen Kadosh, R. Combining Brain Stimulation and Video Game to Promote Long-Term Transfer of Learning and Cognitive Enhancement. *Sci. Rep.* **2016**, *6* (1), 22003. https://doi.org/10.1038/srep22003.

4. Cappelletti, M.; Gessaroli, E.; Hithersay, R.; Mitolo, M.; Didino, D.; Kanai, R.; Cohen Kadosh, R.; Walsh, V. Transfer of Cognitive Training across Magnitude Dimensions Achieved with Concurrent Brain Stimulation of the Parietal Lobe. *J. Neurosci.* **2013**, *33* (37), 14899–14907. https://doi.org/10.1523/JNEUROSCI.1692-13.2013.

5. Cappelletti, M.; Pikkat, H.; Upstill, E.; Speekenbrink, M.; Walsh, V. Learning to Integrate versus Inhibiting Information Is Modulated by Age. *J. Neurosci.* **2015**, *35* (5), 2213–2225. https://doi.org/10.1523/JNEUROSCI.1018-14.2015.

6. Hauser, T. U.; Rotzer, S.; Grabner, R. H.; Mérillat, S.; Jäncke, L. Enhancing Performance in Numerical Magnitude Processing and Mental Arithmetic Using Transcranial Direct Current Stimulation (TDCS). *Front. Hum. Neurosci.* **2013**, *7*. https://doi.org/10.3389/fnhum.2013.00244.

7. Li, L. M.; Leech, R.; Scott, G.; Malhotra, P.; Seemungal, B.; Sharp, D. J. The effect of oppositional parietal transcranial direct current stimulation on lateralized brain functions. *Eur. J. Neurosci*. **2015**, *42* (11), 2904–2914. https://doi:10.1111/ejn.13086

8. Brezis, N.; Bronfman, Z. Z.; Jacoby, N.; Lavidor, M.; Usher, M. Transcranial Direct Current Stimulation over the Parietal Cortex Improves Approximate Numerical Averaging. *J. Cogn. Neurosci.* **2016**, *28* (11), 1700–1713. https://doi.org/10.1162/jocn_a_00991.

9. Hartmann, M.; Singer, S.; Savic, B.; Müri, R. M.; Mast, F. W. Anodal High-Definition Transcranial Direct Current Stimulation over the Posterior Parietal Cortex Modulates Approximate Mental Arithmetic. *J. Cogn. Neurosci.* **2020**, *32* (5), 862–876. https://doi.org/10.1162/jocn_a_01514.

10. Labree, B.; Corrie, H.; Karolis, V.; Didino, D.; Cappelletti, M. Parietal alpha-based inhibitory abilities are causally linked to numerosity discrimination. *Behav. Brain. Res*. **2020**, 387:112564. https://doi:10.1016/j.bbr.2020.112564

11. Grabner, R. H.; Rütsche, B.; Ruff, C. C.; Hauser, T. U. Transcranial Direct Current Stimulation of the Posterior Parietal Cortex Modulates Arithmetic Learning. *Eur. J. Neurosci* **2015**, *42* (1), 1667–1674. https://doi.org/10.1111/ejn.12947.

12. Snowball, A.; Tachtsidis, I.; Popescu, T.; Thompson, J.; Delazer, M.; Zamarian, L.; Zhu, T.; Cohen Kadosh, R. Long-Term Enhancement of Brain Function and Cognition Using Cognitive Training and Brain Stimulation. *Curr. Biol.* **2013**, *23* (11), 987–992. https://doi.org/10.1016/j.cub.2013.04.045.

13. Popescu, T.; Krause, B.; Terhune, D. B.; Twose, O.; Page, T.; Humphreys, G.; Cohen Kadosh, R. Transcranial Random Noise Stimulation Mitigates Increased Difficulty in an Arithmetic Learning Task. *Neuropsychologia* **2016**, *81*, 255–264. https://doi.org/10.1016/j.neuropsychologia.2015.12.028

14. Mosbacher, J. A.; Halverscheid, S.; Pustelnik, K.; Danner, M.; Prassl, C.; Brunner, C.; Vogel, S. E.; Nitsche, M. A.; Grabner, R. H. Theta Band Transcranial Alternating Current Stimulation Enhances Arithmetic Learning: A Systematic Comparison of Different Direct and Alternating Current Stimulations. *Neuroscience*. **2021,** 477:89–105. https://doi:10.1016/j.neuroscience.2021.10.006

15. Clemens, B.; Jung, S.; Zvyagintsev, M.; Domahs, F.; Willmes, K. Modulating Arithmetic Fact Retrieval: A Single-Blind, Sham-Controlled TDCS Study with Repeated FMRI Measurements. *Neuropsychologia* **2013**, *51* (7), 1279–1286. https://doi.org/10.1016/j.neuropsychologia.2013.03.023

16. Pasqualotto, A. Transcranial Random Noise Stimulation Benefits Arithmetic Skills. *Neurobiol. Learn. Mem.* **2016**, *133*, 7–12. https://doi.org/10.1016/j.nlm.2016.05.004.

17. Kasahara, K.; Tanaka, S.; Hanakawa, T.; Senoo, A.; Honda, M. Lateralization of Activity in the Parietal Cortex Predicts the Effectiveness of Bilateral Transcranial Direct Current Stimulation on Performance of a Mental Calculation Task. *Neurosci. Lett.* **2013**, *545*, 86–90. https://doi.org/10.1016/j.neulet.2013.04.022.

18. Sarkar, A.; Dowker, A.; Cohen Kadosh, R. Cognitive Enhancement or Cognitive Cost: Trait-Specific Outcomes of Brain Stimulation in the Case of Mathematics Anxiety. *J. Neurosci.* **2014**, *34* (50), 16605–16610. https://doi.org/10.1523/JNEUROSCI.3129-14.2014.

19. Klein, E.; Mann, A.; Huber, S.; Bloechle, J.; Willmes, K.; Karim, A. A.; Nuerk, H. C.; Moeller, K. Bilateral bi-cephalic tDCS with two active electrodes of the same polarity modulates bilateral cognitive processes differentially. *PLoS ONE* **2013**, *8* (8). https://doi:10.1371/annotation/c7fb111c-b586-4495-a55c-f779d1197810]

20. Artemenko, C.; Moeller, K.; Huber, S.; Klein, E. Differential influences of unilateral tDCS over the intraparietal cortex on numerical cognition. *Front. Hum. Neurosci*. **2015,** 9:110. https://doi:10.3389/fnhum.2015.00110

21. Bieck, S. M.; Artemenko, C.; Moeller, K.; Klein, E. Low to No Effect: Application of Trns During Two-Digit Addition. *Front. Neurosci*. **2018**, 12:176. https://doi:10.3389/fnins.2018.00176

22. Pope, P. A.; Brenton, J. W.; Miall, R. C. Task-Specific Facilitation of Cognition by Anodal Transcranial Direct Current Stimulation of the Prefrontal Cortex. *Cereb. Cortex* **2015,** 25 (11), 4551–4558. https://doi:10.1093/cercor/bhv094

23. Rütsche, B.; Hauser, T. U.; Jäncke, L.; Grabner, R. H. When Problem Size Matters: Differential Effects of Brain Stimulation on Arithmetic Problem Solving and Neural Oscillations. *PLoS ONE* **2015**, *10* (3), e0120665. https://doi.org/10.1371/journal.pone.0120665.

24. Mosbacher, J. A.; Brunner, C.; Nitsche, M. A.; Grabner, R. H. Effects of Anodal TDCS on Arithmetic Performance and Electrophysiological Activity. *Front. Hum. Neurosci.* **2020**, *14*, 17. https://doi.org/10.3389/fnhum.2020.00017.

25. Hauser, T. U.; Rütsche, B.; Wurmitzer, K.; Brem, S.; Ruff, C. C.; Grabner, R. H. Neurocognitive Effects of Transcranial Direct Current Stimulation in Arithmetic Learning and Performance: A Simultaneous TDCS-FMRI Study. *Brain Stimul.* **2016**, *9* (6), 850–858. https://doi.org/10.1016/j.brs.2016.07.007.

26. Krause, B.; Dresler, M.; Looi, C. Y.; Sarkar, A.; Cohen Kadosh, R. Neuroenhancement of High-Level Cognition: Evidence for Homeostatic Constraints of Non-Invasive Brain Stimulation. *J. Cogn. Enhanc.* **2019**, *3* (4), 388–395. https://doi.org/10.1007/s41465-019-00126-7.
